# Comparative Proteome Analysis of Multi-Layer Cocoon of the Silkworm, *Bombyx mori*

**DOI:** 10.1371/journal.pone.0123403

**Published:** 2015-04-10

**Authors:** Yan Zhang, Ping Zhao, Zhaoming Dong, Dandan Wang, Pengchao Guo, Xiaomeng Guo, Qianru Song, Weiwei Zhang, Qingyou Xia

**Affiliations:** State Key Laboratory of Silkworm Genome Biology, Southwest University, Chongqing 400715, China; Uppsala University, SWEDEN

## Abstract

*Bombyx mori* cocoon has a multi-layer structure that provides optimal protection for silkworm pupa. Research on the mechanical properties of the multi-layer structure revealed structure-property relationships of the cocoon. Here, we investigated the protein components of the *B*. *mori* cocoon in terms of its multi-layer structure. Liquid chromatography-tandem mass spectrometry identified 286 proteins from the multiple cocoon layers. In addition to fibroins and sericins, we identified abundant protease inhibitors, seroins and proteins of unknown function. By comparing protein abundance across layers, we found that the outermost layer contained more sericin1 and protease inhibitors and the innermost layer had more seroin1. As many as 36 protease inhibitors were identified in cocoons, showing efficient inhibitory activities against a fungal protease. Thus, we propose that more abundant protease inhibitors in the outer cocoon layers may provide better protection for the cocoon. This study increases our understanding of the multi-layer mechanism of cocoons, and helps clarify the biological characteristics of cocoons. The data have been deposited to the ProteomeXchange with identifier PXD001469.

## Introduction

Many insect larvae spin silky cocoons for pupation. Cocoons protect pupae from predation and microbial degradation and prevent dehydration during metamorphosis [1–5]. The most well-characterized cocoon is from the domestic silkworm, *Bombyx mori*. The cocoon has been used for textile material for around 5000 years. Silkworms spin cocoons at the final larval stage. After finding a suitable place, silkworm larvae construct a loose scaffold silk and then spin cocoons that are firmly attached to a substrate by scaffold silk [6, 7].

The cocoon silk of *B*. *mori* is a natural polymer with a length of 1000~1500 m. It is mainly composed of two threads (fibroins) bonded by adhesive proteins (sericins) [8]. Besides fibroins and sericins, proteins with low molecular weights such as seroins and protease inhibitors are found in *B*. *mori* cocoon extracts [4, 9, 10]. Recently, we identified 169 proteins in cocoon silk using shotgun liquid chromatography-tandem mass spectrometry (LC-MS/MS) [11]. In addition to fibroins and sericins, other proteins were also found, including enzymes, protease inhibitors, and proteins of unknown functions [11]. Compared with scaffold silk, cocoon silk contains fewer sericins, protease inhibitors and enzymes, reflecting its different function [11].

The *B*. *mori* cocoon has a multi-layer structure with fewer fibers connecting the layers than are aligned in the individual layers. Interlayer bonding is much weaker than intralayer bonding [12]. From the outer layer toward the inner layer, both elastic modulus and tensile strength increase, allowing the cocoon to resist outside forces [13, 14]. The microstructure of the cocoon layer was revealed by scanning electron microscope (SEM) and suggests that the better mechanical properties of the inner layer are due to its thinner fiber diameter, more dense silk distribution and lower porosity [13, 14]. Density and porosity of cocoon layers are correlated with protein components. Both SEM and Fourier transform infrared spectra of cocoon layers show that the inner layer has less sericin than the outer layer [14]. The reduced sericin in the inner layer efficiently bonds the fibroins, but the increased sericin in the outer layer does not result in additional bonding between fibers [14].

The cocoon layers have different microstructures and mechanical properties to protect the pupa. However, whether the layers also have distinct protein components to support pupa at the biochemical level is unknown. Here, we employed LC-MS/MS to investigate the protein components of *B*. *mori* cocoon in its multiple layer structure. We believe that the results will be particularly useful for gaining a deeper understanding of the multi-layer structure and function of *B*. *mori* cocoons.

## Materials and Methods

### Materials

The Chinese silkworm strain DaZao, provided by State Key Laboratory of Silkworm Genome Biology, was reared on mulberry leaves at a stable temperature of 25°C. Silkworms spun complete cocoons at the end of the fifth larval instar stage. Cocoons were split into five layers after removing amorphous scaffold silk from the surface.

### Sample Preparation

Layers of cocoon silks (10 mg) were dissolved in 0.5 mL 9 M LiSCN with vortexing for 2 h. Solubilized proteins were recovered by centrifugation (12,000 g, 10 min, 4°C). Equal amounts of silk proteins (5 μL) were separated on 12.5% (w/v) polyacrylamide gels and visualized by silver nitrite staining. Silk proteins were digested according to the Filter Aided Sample Preparation (FASP) method [15] and placed in an ultrafiltration tube (MWCO 10,000, Millipore, USA), washed three times with 8 M urea using centrifugation at 12,000 g, 4°C for 20 min, reduced with 15 mM dithiothreitol for 120 min at 37°C and alkylated with 50 mM iodoacetamide for 60 min in the dark. Samples were washed three times with 8 M urea and three times with 50 mM NH_4_HCO_3_ and proteins were digested with trypsin at a weight ratio of 1:50 (trypsin:protein) for 20 hours at 37°C. Tryptic peptides were recovered by centrifugation, lyophilized, and resuspended in 80 μL 0.1% formic acid.

### Mass Spectrometry

Tryptic peptides (2 μL) separated on a Thermo Fisher Scientific EASY-nLC 1000 system using a Thermo Fisher Scientific EASY-Spray column (C18, 2 μm, 100 Å, 50 μm × 15 cm) with a 140 min gradient of 2 min 3%~8% Buffer B (100% acetonitrile, 0.1% formic acid), 100 min 8%~20% Buffer B, 10 min 20%~30% Buffer B, 5 min 30% ~70% Buffer B, 3 min 70%~90% Buffer B, and 20 min 90% Buffer B. Peptides were analyzed using a Thermo Scientific Q Exactive mass spectrometer in data-dependent mode with an automatic switch between MS and MS/MS scans using a top 20 method. Instrument parameters were: resolution 70,000 for full MS scan and 17,500 for MS^2^ scan, automatic gain control target 3e6 for full scan and 1e6 for MS^2^, maximum ion injection time 20 ms for full MS scan and 60 ms for MS^2^ scan.

### Protein Identification

Mass spectra raw data were analyzed using MaxQuant software (version 1.3.0.1) [16]. The MaxQuant searches were executed against an integrated silkworm proteome database containing 35,379 protein sequences from NCBI and silkDB (downloaded on October 17, 2013, Supplementary S1 Dataset). Peptide searches were performed with Andromeda search algorithms [17] using search parameters: maximum of two missed cleavages permitted, carbamidomethyl cysteine as fixed modification, and oxidation (methionine) and acetylation (N-terminus proteins) as variable modifications. Mass tolerance was 20 ppm for first search and 6 ppm for main search. False discovery rate was 0.01 for both proteins and peptides, which had a minimum length of 6 amino acids. The mass spectrometry proteomics data have been deposited to the ProteomeXchange Consortium [18] via the PRIDE partner repository with the dataset identifier PXD001469. Identified peptides were combined and reported as protein groups. A minimum of one unique peptide was required for an identified protein. All common contaminants and reverse hits were removed. Identified peptides and proteins are in S1 Table and S2 Table, respectively.

### Protein Quantitation

The intensity-based absolute quantification (iBAQ) algorithm in MaxQuant was used to compare abundances of different proteins within a single sample; the label-free quantification (LFQ) algorithm in MaxQuant was employed to compare the abundances of protein abundances among different samples. Relative intensity of fibroin heavy chain to other proteins was assumed to be essentially the same in all silks and was normalized as 100,000. Relative intensity of proteins was normalized to fibroin heavy chain [11]. Estimates of protein intensity are in S2 Table.

### Protein Annotation

Blast2GO software (version 2.6.6) was used to annotate molecular functions of cocoon proteins [19]. BLASTp searches were first done against a non-redundant database and further analyses included gene ontology (GO) and enzyme code (EC) annotations. Default Blast2GO settings were used at each step. SignalP 4.0 Server was used to predict the presence of the signal peptides (http://www.cbs.dtu.dk/services/SignalP/) [20]. Pfam was used to predict domain architecture (http://pfam.janelia.org/). Amino acid sequences were aligned using ClustalX 1.83 [21]. Alignments were manually modified using GeneDoc [22].

### Degradation of fibroins by proteases

To extract fibroins, cocoons were cut into small pieces and dissolved in 8 M urea (10 min, 80°C) to remove sericins. Remaining insoluble silk was washed twice with 8 M urea and recovered by centrifugation (12,000 g, 10 min, 4°C) and dissolved in 9 M LiSCN with vortexing for 2 h. Fibroin degradation experiments used four proteases: trypsin, chymotrypsin, elastase, and protease K. Protease (0.05 μg) and fibroin (50 μg) were mixed in a microcentrifuge tube and incubated for 1 h at 37°C. Reaction products (10 μL) were separated on 12.5% (w/v) polyacrylamide gels and visualized by coomassie brilliant blue staining.

### Protease inhibitor activity assay

To extract protease inhibitors, cocoons were cut into small pieces and immersed in 100 mM Tris-HCl buffer (pH 7.4) with stirring overnight at 4°C. Solubilized cocoon proteins were recovered by filtration and centrifugation (12,000 g, 10 min, 4°C). For protease inhibitor activity assays, four proteases were used as target proteases: trypsin (Sigma, T1426), chymotrypsin (Sigma, C4129), elastase (Sigma, 45124), and protease K (Roche, 11060325). Protease was added to 100 mM Tris-HCl buffer (pH 7.4) in a microcentrifuge tube followed by addition of cocoon proteins. After 15 min of pre-incubation at room temperature, casein was added and incubated for 1 h at 37°C. A 200 μL reaction system contained 0.16 μg protease, 4 μg cocoon protein and 80 μg casein substrate. For controls, 100 mM Tris-HCl buffer (pH 7.4) was used instead of protease, cocoon proteins or casein. Equal amounts of protein mixture (10 μL) were separated on 12.5% (w/v) polyacrylamide gels and visualized by coomassie brilliant blue staining.

## Results

### Proteome identification of cocoon layers

**Fig 1:**
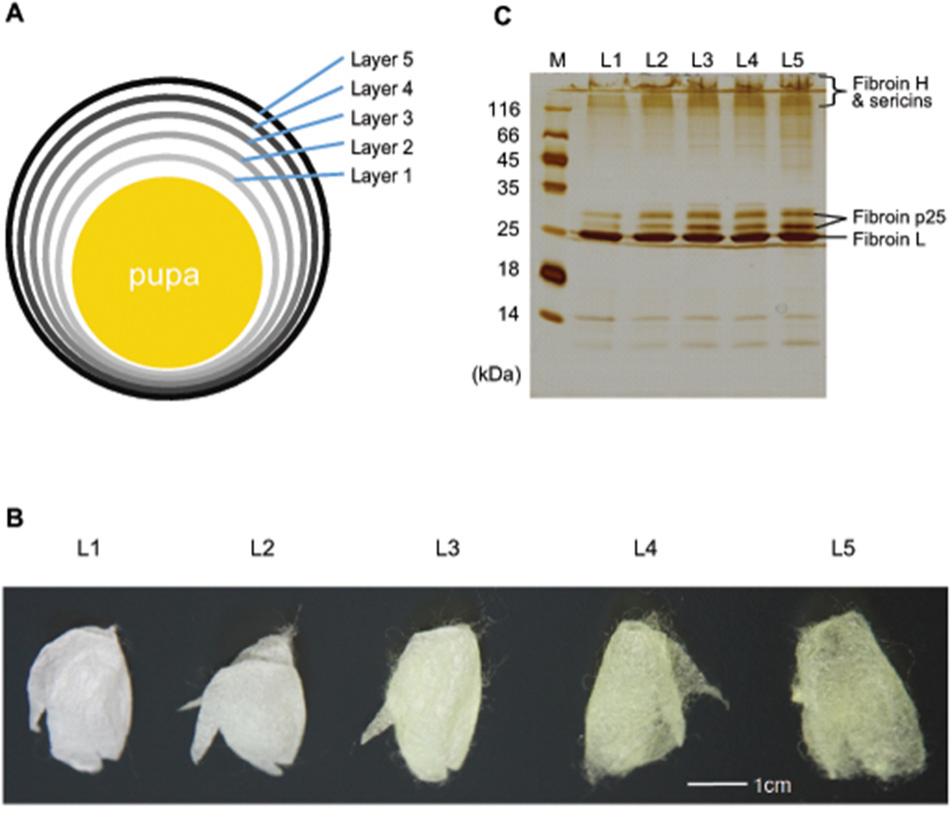
Five cocoon layers of *B*. *mori*. (A) Schematic representation of cocoon layers; (B) Photos of layers; (C) Silver stain of SDS-PAGE of proteins from layers. From the inner layer towards the outer layer, the five cocoon layers were named as layer1~layer5 (L1~ L5), respectively.

Cocoons were split into five layers and named as layer 1~5 from inner to outer (Fig 1A and 1B). Each layer showed a similar protein profile by SDS-PAGE (Fig 1C). Intensely stained bands were annotated according to previous reports [23] and were mainly fibroins (heavy chain, light chain and p25) and sericins. For comparison of the silk proteome from different cocoon layers, LC−MS/MS analyses were performed with three replicates. Combining the data, we identified 2270 tryptic peptides (S1 Table). In the combined dataset, 286 proteins were identified and 266 (93%) had two or more unique peptides (S2 Table). Twenty proteins were identified by only one peptide, and for these we have provided the annotated MS/MS spectrum (S1 Fig). When comparing identifications from the three repeat analyses, 71.4%~80.5% of proteins were identified in all experiments. To assess the completeness of our dataset, we compared it against our previous results [11]. We found that 135 previously identified cocoon proteins (80%) were contained in the new dataset. One hundred fifty novel cocoon proteins were reported for the first time in this study. The identification of new cocoon proteins could be due to two possible reasons: a larger sample amount (five layer samples × triplicates) and a new database with more protein sequences. By layer, we identified 241 proteins in layer 1, 133 in layer 2, 138 in layer 3, 162 in layer 4, and 166 proteins in layer 5. Of these, 89 were specific to layer 1 and 13 to layer 5, while 106 were common to all layers (S2 Table).

Based on annotated molecular functions, 286 cocoon proteins were classified into 10 categories: enzymes (120), binding and transport proteins (50), proteins of unknown functions (40), protease inhibitors (36), extracellular matrix proteins (17), protein synthesis related proteins (9), cytoskeletal proteins (6), fibroins (3), sericins (3) and seroins (2) (S2 Table). The 210 proteins (73.4%) have a predicted signal peptide (S2 Table), suggesting that they are secreted proteins from the silk gland cells to the gland cavity, where they make the silk fibers.

### Estimation and comparison of protein abundance among five cocoon layers

**Fig 2:**
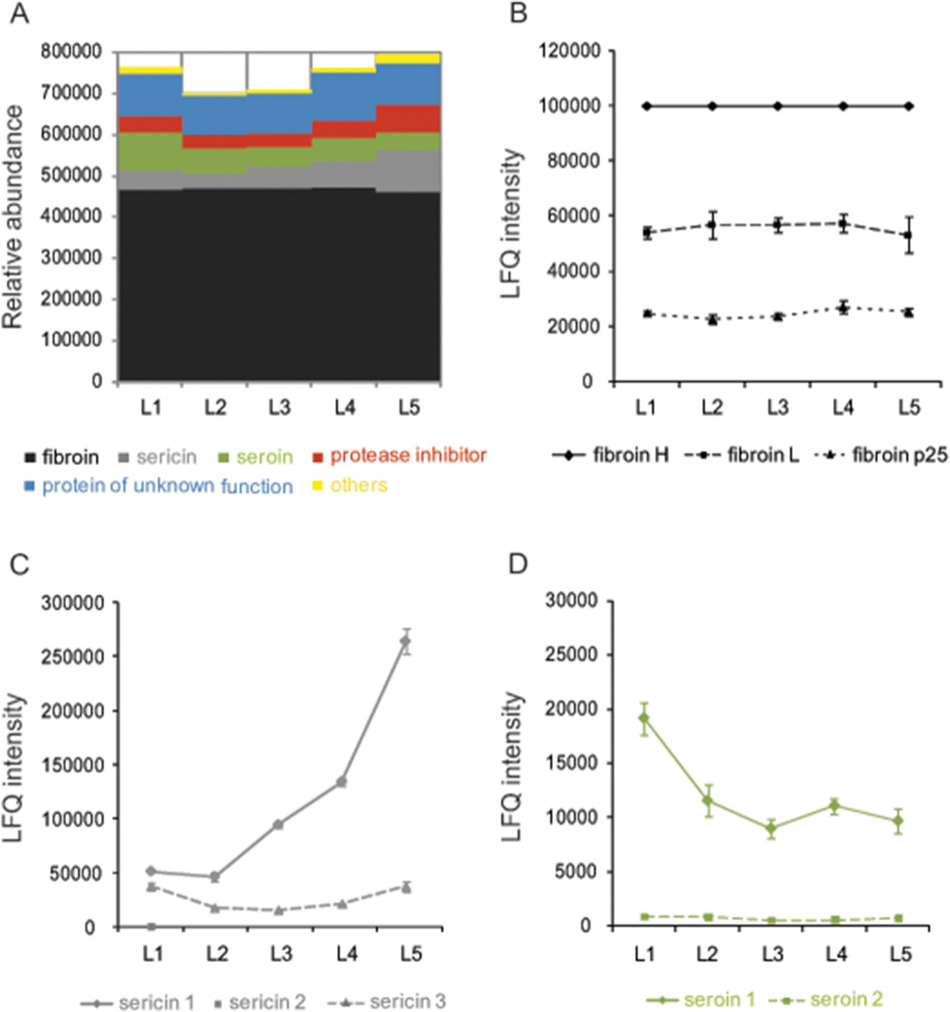
Relative abundance of proteins from functional categories in five cocoon layers. Molar abundance of proteins was estimated with iBAQ intensities (A). Molar abundances of fibroins (B), sericins (C) and seroins (D) were compared among five cocoon layers by LFQ intensities.

To describe the protein content of each cocoon layer, we estimated the relative molar abundance of proteins using the iBAQ algorithm (Fig 2A and S2 Table). Fibroins had the highest molar abundance in all cocoon layers (58.1%~67.0%). Proteins of unknown function had the second highest molar abundance (12.5%~15.3%). Also abundant were sericins (5.1%~12.9%), seroins (5.5%~12.0%), and protease inhibitors (4.6%~8.2%). Other proteins made up about 0.9%~2.9% of the total molar abundance.

**Fig 3:**
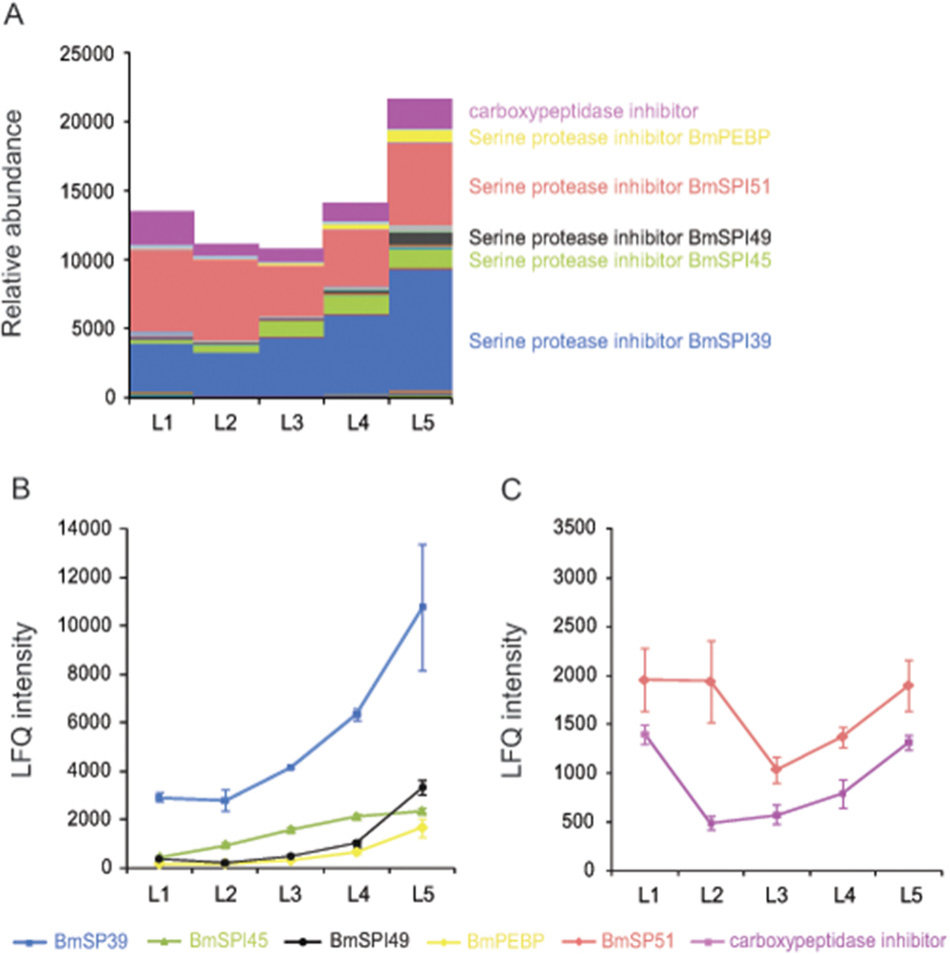
Relative abundance of protease inhibitors in five cocoon layers. Molar abundance of proteins was estimated with iBAQ intensities (A). Six major protease inhibitors were compared among layers by LFQ intensities. Four showed the highest abundance in the outermost layer; the other two showed high abundance in outermost and innermost layers. Proteins labeled "BmSPI" are nomenclature of *B*. *mori* serine protease inhibitors identified by Zhao et al. (2012).

By comparing protein abundance differences among cocoon layers, we found that all the three fibroins were the same (Fig 2B). From the inner layers towards the outer layers, sericin1 increased, sericin3 decreased and then increased and sericin2 showed very low abundance or absence in all cocoon layers (Fig 2C). Two major small molecular seroins are known to be antimicrobial proteins [4, 9, 10]. Seroin1 had the highest abundance in the innermost layer and seroin2 had low abundance with slight differences in the cocoon layers (Fig 2D). Protease inhibitors showed the highest abundance in the outermost layer when compared with other layers, with six protease inhibitors accounting for 89.3%~94.2% of the molar abundance: a carboxypeptidase inhibitor and the five serine protease inhibitors BmPEBP, BmSPI39, BmSPI45, BmSPI49, and BmSPI51 (Fig 3A and Table 1). BmPEBP, BmSPI39, BmSPI45 and BmSPI49 increased from the inner to the outer layers (Fig 3B), whereas the carboxypeptidase inhibitor and BmSPI51 decreased and then increased from the inner to the outer layers (Fig 3C).

**Table 1:** Identification of protease inhibitors in five cocoon layers.

Annotated name	silkDB No.	GeneBank No.	MW [kDa]	Length of signal peptides
serine protease inhibitor serpin type BmSPI2	BGIBMGA007720	gi|112983210	38.7	—
serine protease inhibitor serpin type BmSPI3	BGIBMGA010212	gi|114051043	51.6	22
serine protease inhibitor serpin type BmSPI4	BGIBMGA013852	gi|112982980	46.3	17
serine protease inhibitor serpin type BmSPI5	BGIBMGA013849	gi|112984548	44.4	16
serine protease inhibitor serpin tyep BmSPI9	BGIBMGA001983	gi|14028769	41.9	16
serine protease inhibitor serpin type BmSPI16	BGIBMGA003292	gi|195972034	44.4	20
serine protease inhibitor serpin type BmSPI18	—	gi|226342898	44.0	20
serine protease inhibitor serpin type BmSPI22	—	gi|195972046	44.0	21
serine protease inhibitor serpin type BmSPI28	—	gi|226342914	40.3	—
serine protease inhibitor serpin type BmSPI24	BGIBMGA008829	gi|512908375	44.9	20
serine protease inhibitor serpin type	—	gi|113052	41.5	16
serine protease inhibitor TIL type BmSPI38	BGIBMGA009094	gi|512898433	8.8	22
serine protease inhibitor TIL type BmSPI39	BGIBMGA009092	gi|512898429	22.7	24
serine protease inhibitor TIL type BmSPI40	BGIBMGA009091	gi|512898425	9.7	23
serine protease inhibitor TIL type BmSPI41	BGIBMGA009093	—	5.9	—
serine protease inhibitor TIL type BmSPI45	BGIBMGA006235	gi|512903709	43.2	13
serine protease inhibitor TIL type BmSPI46	BGIBMGA004727	—	30.0	18
serine protease inhibitor TIL type BmSPI47	BGIBMGA004728	—	80.3	—
serine protease inhibitor TIL type BmSPI49	BGIBMGA010889	gi|512898143	62.4	39
serine protease inhibitor TIL type	BGIBMGA004869	—	9.6	19
serine protease inhibitor TIL type	—	gi|512898139	14.3	19
serine protease inhibitor TIL type	BGIBMGA010891	gi|512897973	19.2	18
serine protease inhibitor TIL type	—	gi|512898151	14.1	—
serine protease inhibitor TIL type	—	gi|512938831	7.0	—
serine protease inhibitor kunitz type BmSPI50	—	gi|27549393	9.6	23
serine protease inhibitor kunitz type BmSPI51	—	gi|19070651	8.4	21
serine protease inhibitor kunitz type	—	gi|14028771	9.6	23
serine protease inhibitor kunitz type		gi|512894053	8.9	21
serine protease inhibitor kazal type BmSPI60	BGIBMGA011573	gi|87248517	10.6	18
serine protease inhibitor amfpi type BmSPI69	BGIBMGA008205	gi|37654364	10.7	19
serine protease inhibitor ITI type BmSPI70	BGIBMGA007558	gi|512899354	99.1	22
serine protease inhibitor pacifastin type BmSPI73	—	gi|512910930	15.0	—
serine protease inhibitor PEBP type	BGIBMGA013261	gi|512891705	23.1	26
serine protease inhibitor A2M type	BGIBMGA013565	gi|512909835	155.9	21
cysteine proteinase inhibitor (Inhibitor_I29)	BGIBMGA007039	gi|112983070	12.2	19
carboxypeptidase inhibitor (Inhibitor_I68)	—	gi|164448666	12.6	15

### Prediction and detection of inhibitory activities of protease inhibitors

**Fig 4:**
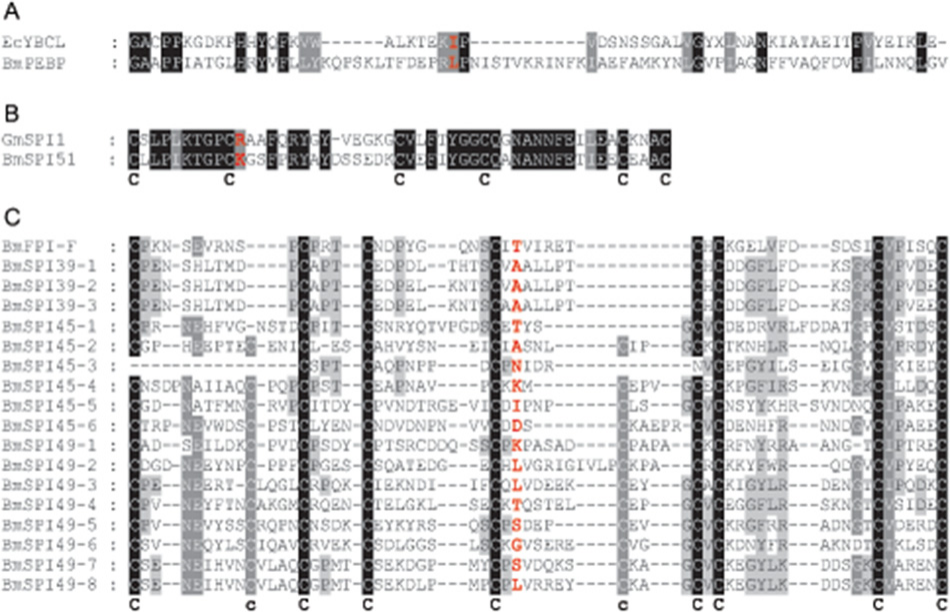
Sequence alignments of main cocoon protease inhibitors with known inhibitors: YBCL from *Escherichia coli*, SPI1 from *Galleria mellonella*, and FPI-F from *Bombyx mori*. (A) PEBP-type protease inhibitors; (B) kunizt-type protease inhibitors; (C) TIL-type protease inhibitors. Alignments were by ClustalX 1.83 with default parameters and shading using GeneDoc. Black, identical residues; gray, similar residues; red, predicted P1 position; C, conserved cysteine position.

By predicting the domains of protease inhibitors, we found that the 6 major protease inhibitors had 4 different domains (Table 1). By sequence alignment with reported homologous protease inhibitors [4, 24, 25], we found the P1 position was Leu in BmPEBP, Lys in BmSPI51 and Ala in BmSPI39 (Fig 4A, 4B and 4C). The specificity of each protease inhibitor mainly depends on P1 residue at the reactive site [26]. Therefore, specific inhibitory activities might be against chymotrypsin-like enzymes for BmPEBP, trypsin-like enzymes for BmSPI51 and elastase-like enzymes for BmSPI39 (Fig 4A, 4B and 4C). Both BmSPI45 and BmSPI49 have multiple tandem TIL domains and varied amino acid residues in the P1 active site (Fig 4C) and thus probably have broad-spectrum inhibitory activities (Fig 4C).

**Fig 5:**
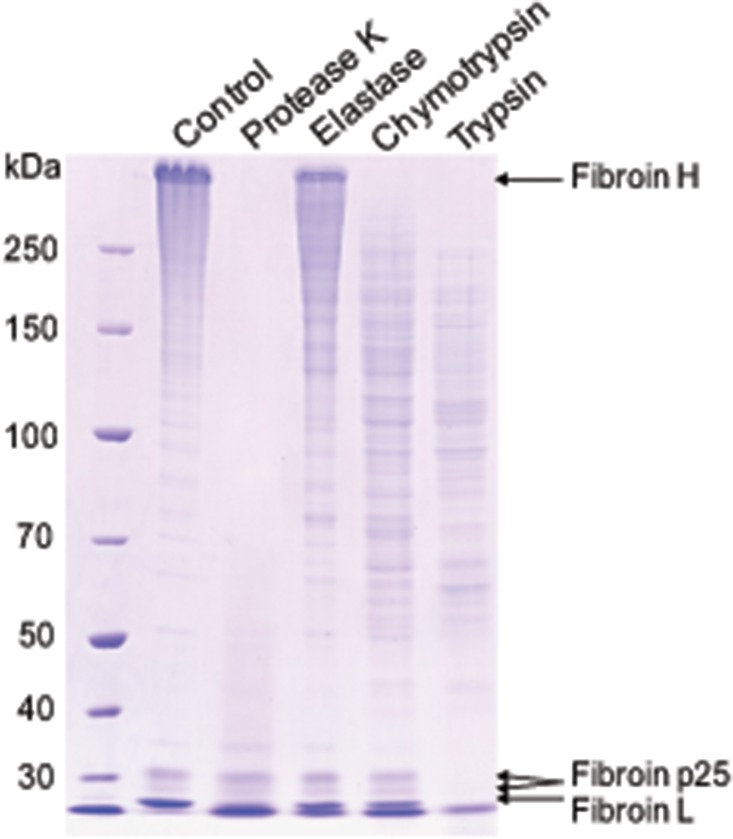
Degradation of fibroins by proteases. Sericin-free fibroins were incubated with trypsin, chymotrypsin, elastase, or protease K for 1 h at 37°C. Proteases were 0.05 μg. Fibroin was 50 μg. Products and controls were separated by SDS-PAGE and stained by coomassie brilliant blue.

**Fig 6:**
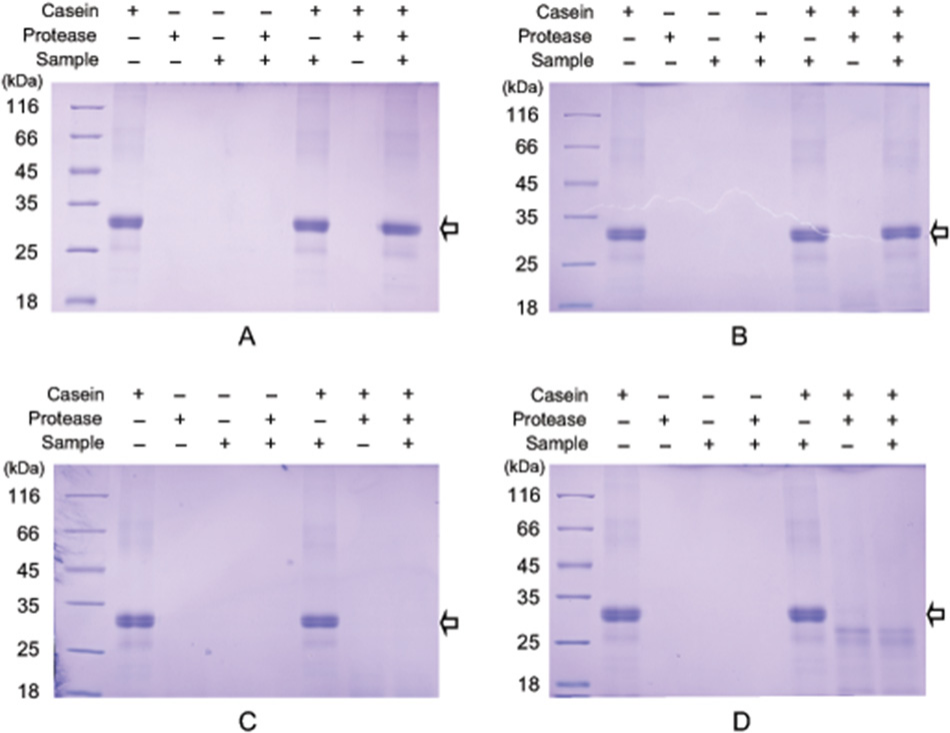
Inhibitory effects of cocoon protease inhibitors on proteases. Extracted water-soluble protein samples from cocoons were incubated with protease K (A), trypsin (B), chymotrypsin (C), and elastase (D) for 15 min at room temperature and casein was added for 1 h at 37°C. Reaction products and controls were separated by SDS-PAGE and stained by coomassie brilliant blue.

By incubating the fibroins with different proteases, we found that fungal protease K showed stronger proteolytic activity against fibroins than animal proteases, including trypsin, chymotrypsin and elastase (Fig 5). This result implied that protease K inhibitors may be more needed than other inhibitors to protect the cocoon. To verify our speculation, casein was used for activity assay as a universal protease substrate in activity assays. Digestion of casein by protease K and trypsin were completely inhibited by cocoon proteins (Fig 6A and 6B), suggesting that cocoon protease inhibitors efficiently inhibited protease K and trypsin. However, cocoon protease inhibitors showed almost no inhibitory activity towards chymotrypsin and elastase (Fig 6C and 6D).

## Discussion

Silkworms spin silk cocoons at the end of the last larval instar stage and become pupae inside. Silkworm pupae are an extremely valuable natural resource rich in protein, fat and other ingredients [27] and are a preferred food for microorganisms, birds and other animals. Once the silkworm becomes a pupa, it cannot eat or move and has little resistance against dangers and a harsh external environment. Therefore, cocoons are an important protection for pupae, but we do not fully understand how cocoons protect pupae. Previous studies determined the outstanding structure and mechanical properties of cocoons [12–14, 28–30]. In this study, we found several cocoon proteins with defenseive functions that had different distributions from the inner to outer layer.

This study found that all three fibroin proteins had a constant abundance in the different cocoon layers. This is consistent with the fact that fibroins are made up of the stable center structure of the silk fiber. However, three sericins showed distinct distributions in different cocoon layers, implying functional differences. Previous studies found that the inner cocoon layers have less sericin than the outer layers [14] and our results further showed that the sericin decrease in the inner layers was due to a decrease in sericin1, which was far abundant than sericin2 and sericin3 in cocoons. The silkworm cocoon is made up of a long silk filament of two fibroin threads (brins) covered with sericins [31, 32]. The combination of fibroins and sericins primarily establishes the cocoon structure. The cocoon contains many proteins of unknown function, some of which have an abundance second only to fibroins and sericins; these include the glycine-rich cell wall structural protein 1.0-like, osiris 9 like-1 and fibroin p25 like-1 (S2 Table). Elucidating the functions of these proteins will promote our understanding of the skeletal construction of cocoons.

In addition to fibroins and sericins, our results found abundant antimicrobial proteins such as seroins and protease inhibitors in cocoons. Two small homologous proteins, seroin1 and seroin2, are found in lepidopteran silks [33, 34]. They were recently described as effective antimicrobial proteins that inhibit bacterial and viral infection of *B*. *mori* [10]. Our study found additional seroin2 in the scaffold silk that covers the cocoon [11] and seroin1 in the innermost cocoon layer, implying differential functions.

Protease inhibitors constitute a large protein group in cocoons. Serine protease inhibitors are the most diverse and abundant protease inhibitors in cocoons. Our prediction results indicated that these inhibitors might inhibit chymotrypsin-like enzymes, trypsin-like enzymes and elastase-like enzymes. Our activity assays suggested that protease inhibitors in cocoons have higher inhibitory activity against trypsin and protease K than other proteases. In fact, six trypsin inhibitors have been found in the cocoons of 64 silkworm strains [35]. Protease K is a broad-spectrum serine protease from the fungus *Tritirachium album* [36, 37] that has ability to digests fibroins, and might be used by fungi to destroy cocoons. This may be why protease K inhibitors are abundant in cocoons. Previous studies found that a kunitz-type inhibitor GmSPI1 and a kazal-type inhibitor GmSPI2 in *Galleria mellonella* cocoons show high inhibitory activities against both fungal protease K and bacterial subtilisin [4]. We found that *B*. *mori* cocoons also contain both kunitz-type and kazal-type inhibitors. In addition, many other types of protease inhibitors were reported in cocoons, including serpin, TIL, amfpi, ITI, pacifastin, PEBP, A2M, Inhibitor_I29, and Inhibitor_I68. Two TIL-type inhibitors FPI-F and BmSPI38 from silkworm had been proved to inhibit microbial proteases [38, 39]. One recent study suggested that two proteinase inhibitors BmSPI38 and BmSPI39 could significantly inhibit the germination of *Beauveria bassiana* conidia [40]. The presence of abundant protease inhibitors in cocoons implies they are important in preventing the cocoon destruction by microbial proteases. Protease inhibitors showed an inhomogeneous distribution in cocoon layers, with more in the outermost layer than in other layers. Scaffold silk covers the cocoon and contains more protease inhibitors than cocoon silk [11]. The gradient distribution of protease inhibitors in different layers allows the cocoon to persist for a long time in the environment and protects the pupa against infection.

This study found that silkworms build cocoons with fibroins and sericins and also with antimicrobial proteins to avoid infection. This finding is helpful for clarifying the biological characteristics of cocoons, and to explore the further application of the cocoon.

## Supporting Information

S1 Fig The annotated tandem mass spectra of 20 proteins with one single unique peptide. (PDF)

S1 Table Identified peptides from the five cocoon layers of *B*. *mori*. Identified proteins are listed by the sequence, length, number of miscleavages, the charge states, Andromeda score, mass and the lowest posterior error probability (PEP) from among multiple analyses. The PEP is a measure of the probability of a false hit derived from the peptide’s score and its length.
(XLS)

S2 Table Identified proteins from the five cocoon layers of *B*. *mori*. Identified proteins are listed by functional category, accession number in silkDB and Genebank, annotated name, gene ontology annotation, molecular weight, enzyme commission number, the number of peptides and sequence coverage. The iBAQ (intensity-based absolute quantification) and LFQ (label-free quantification) intensities are listed sample by sample.
(XLS)

S1 Dataset The database with 35,379 protein sequences. (TXT)
