# Supplementary figures and images for: Comparative Proteome Analysis of Multi-Layer Cocoon of the Silkworm, Bombyx mori
Source: PLoS One. 2015 Apr 10;10(4):e0123403. doi: 10.1371/journal.pone.0123403 (PMC4393245; doi:10.1371/journal.pone.0123403)

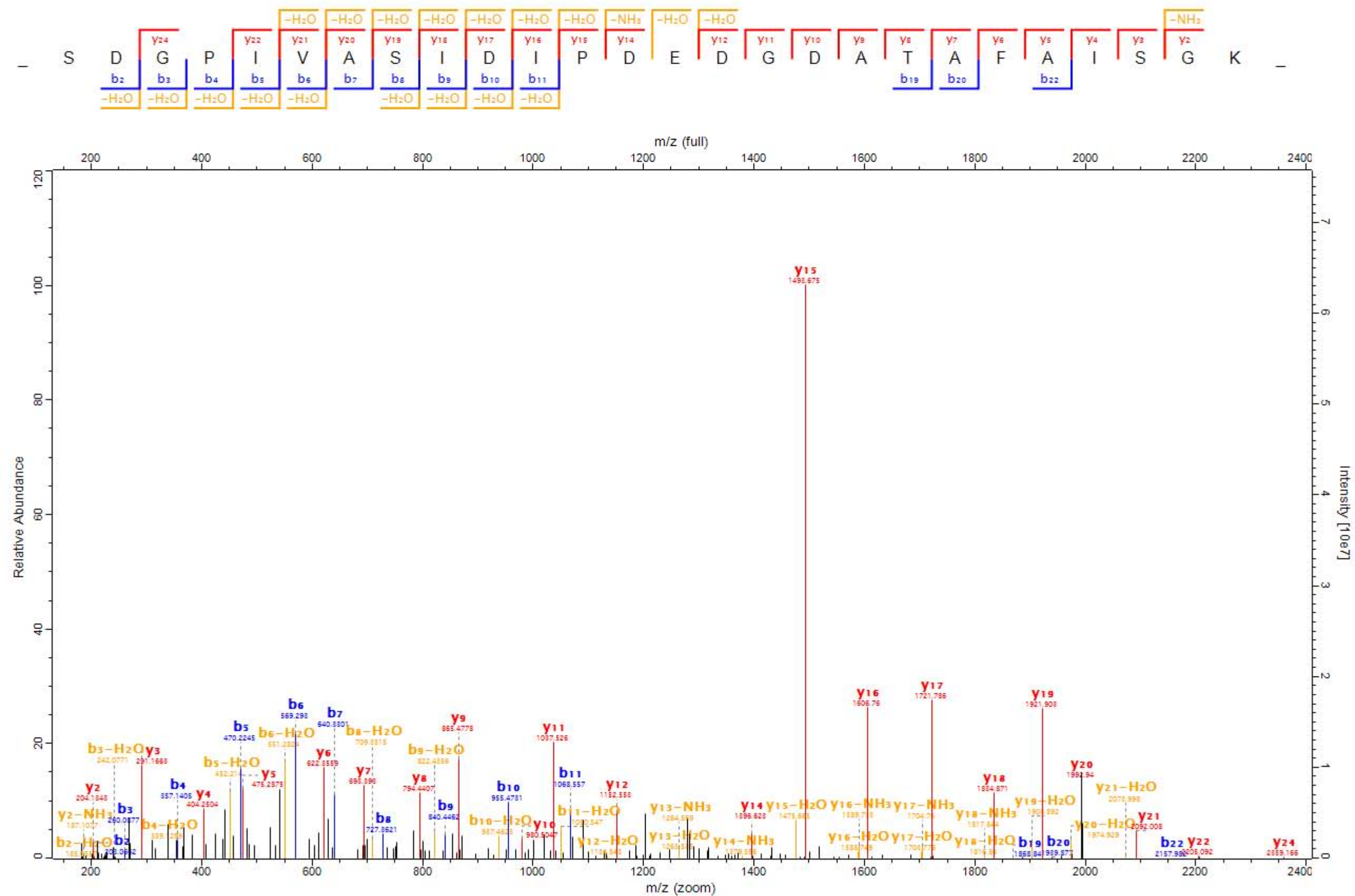

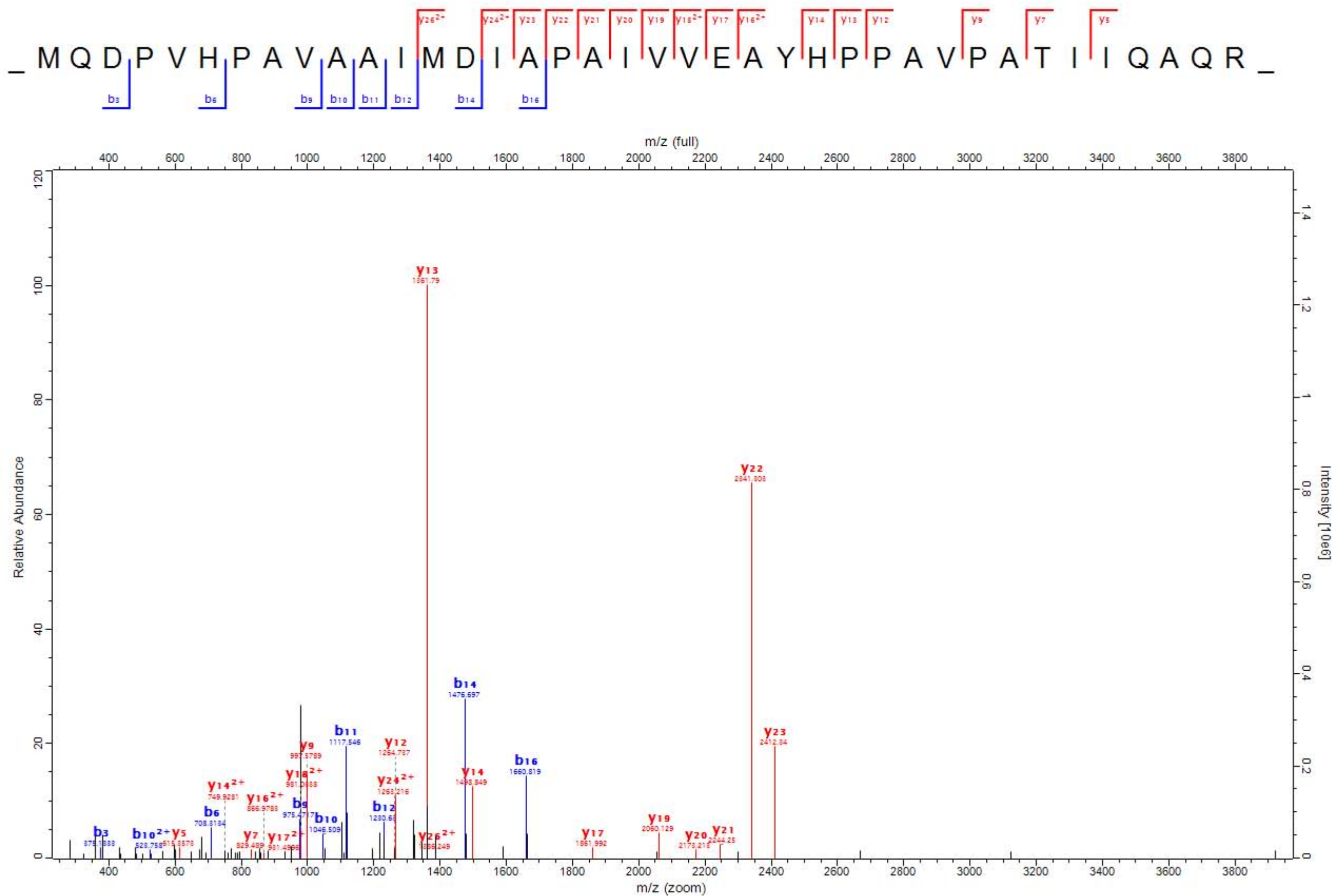

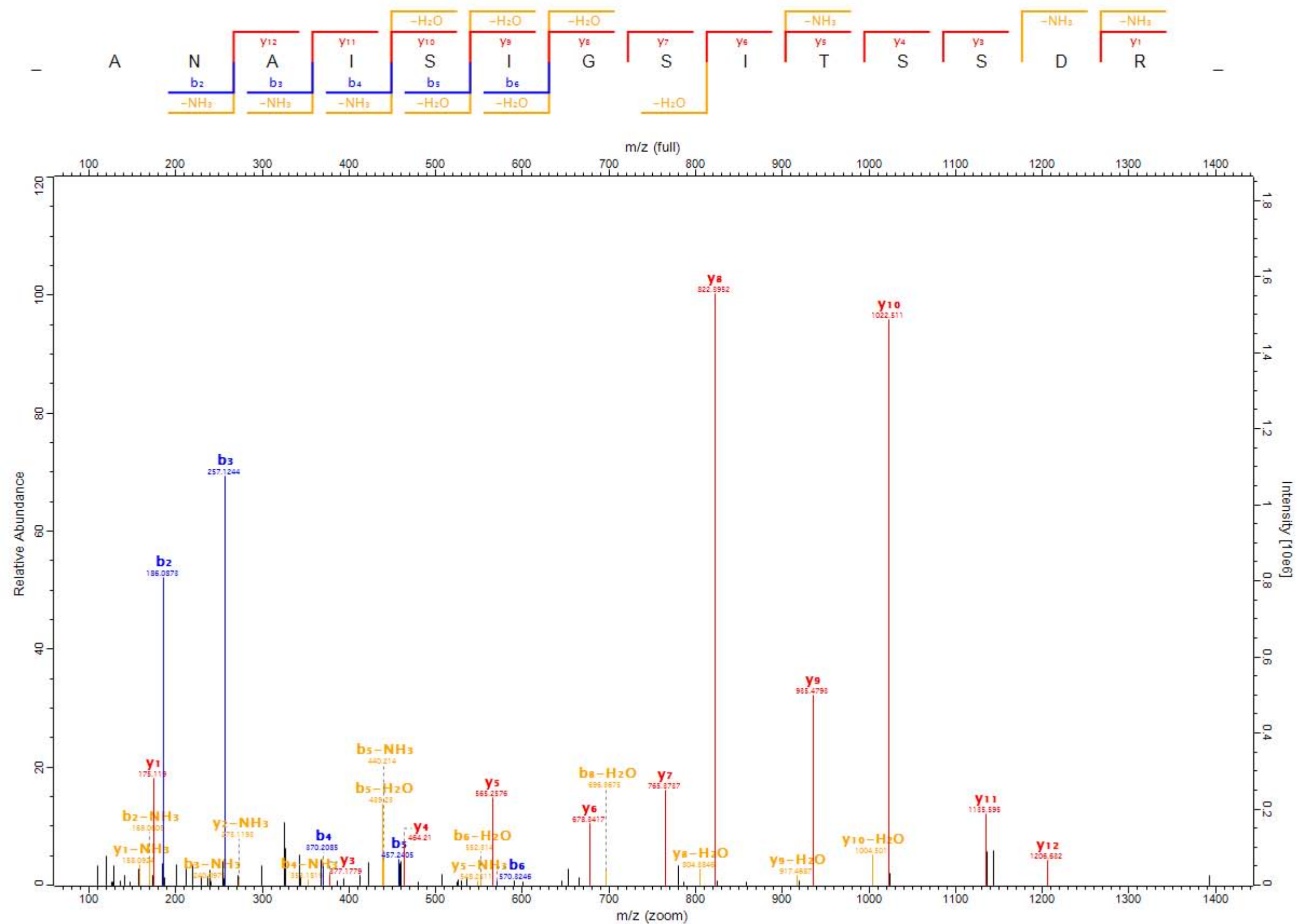

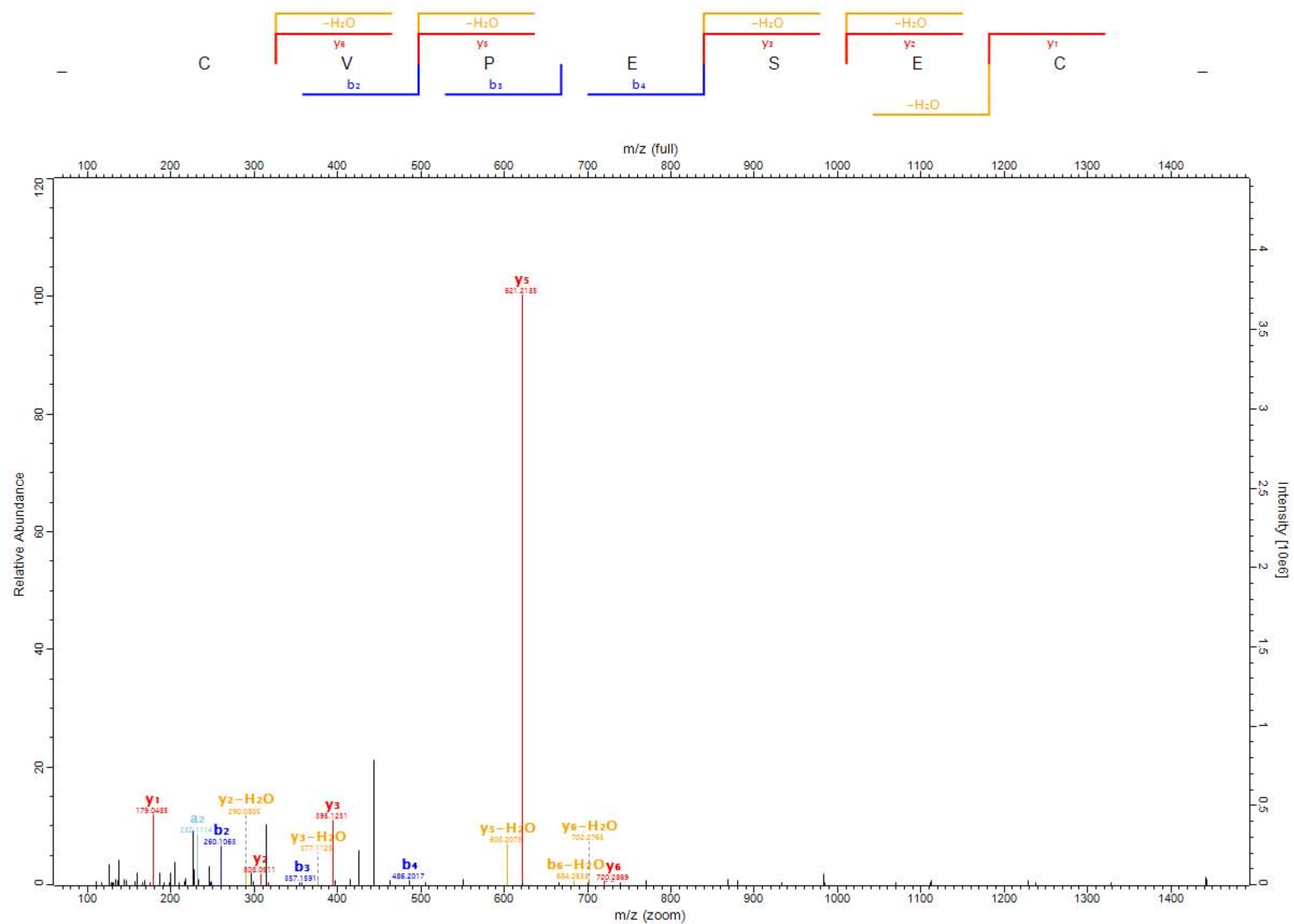

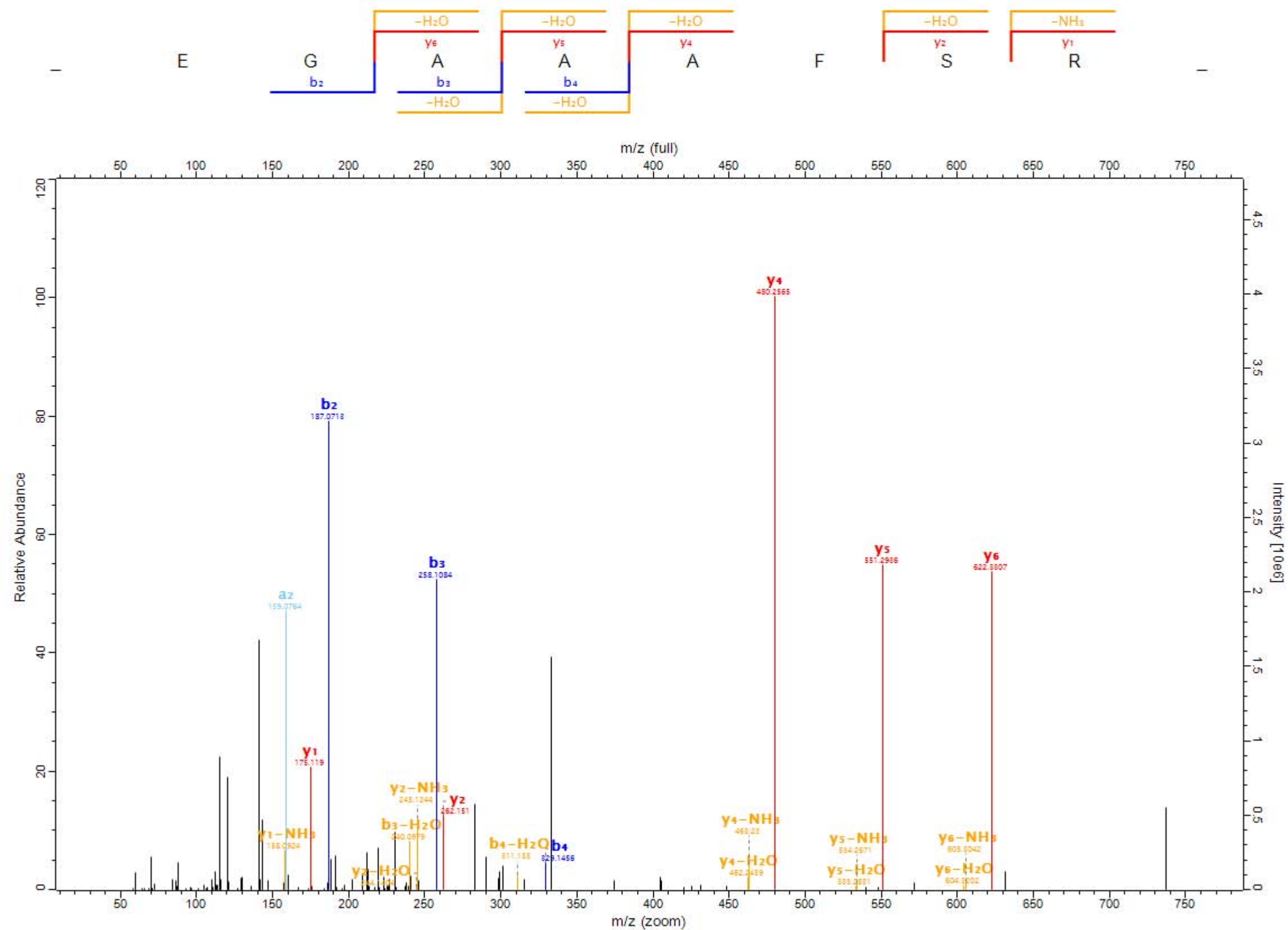

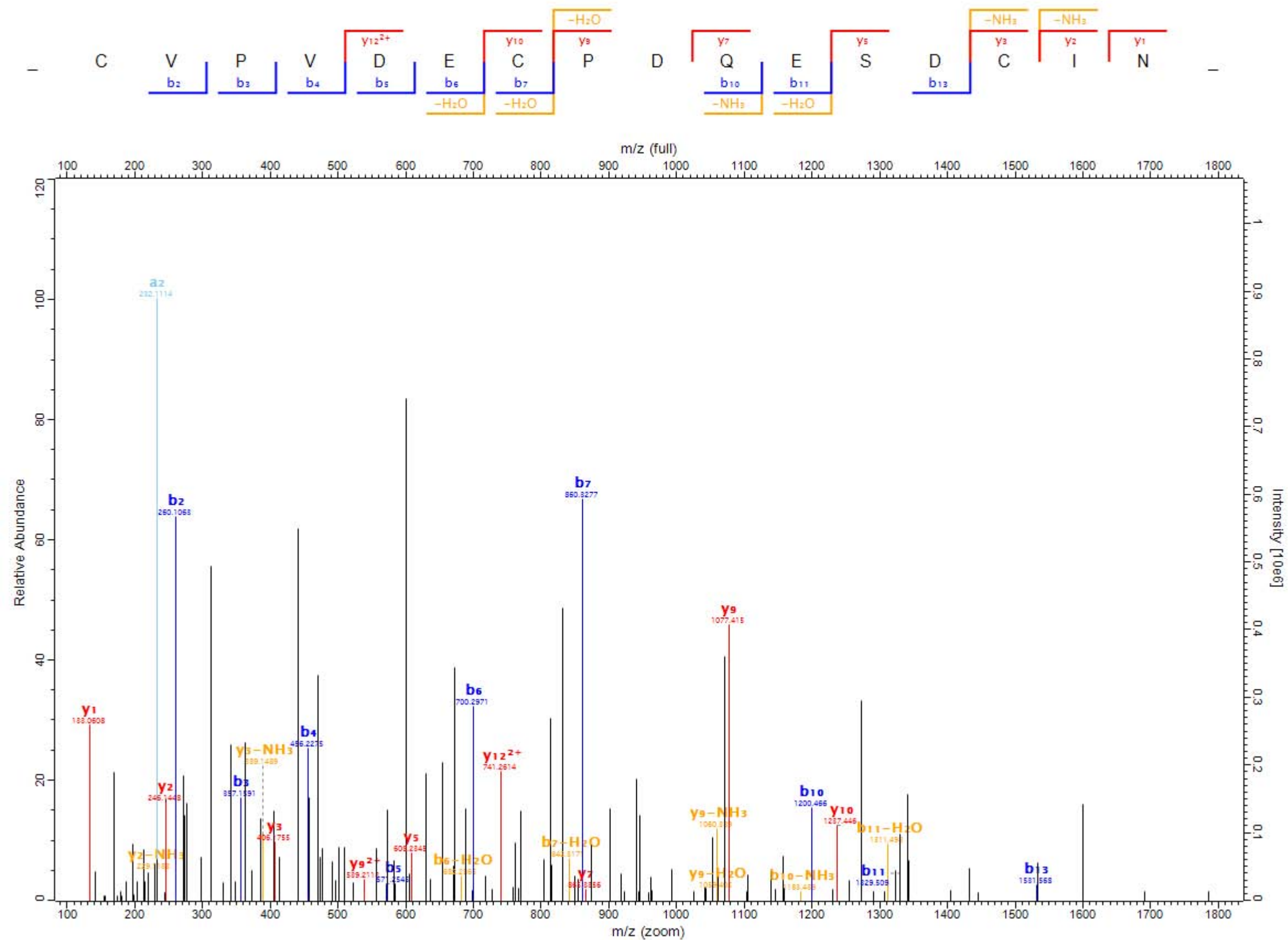

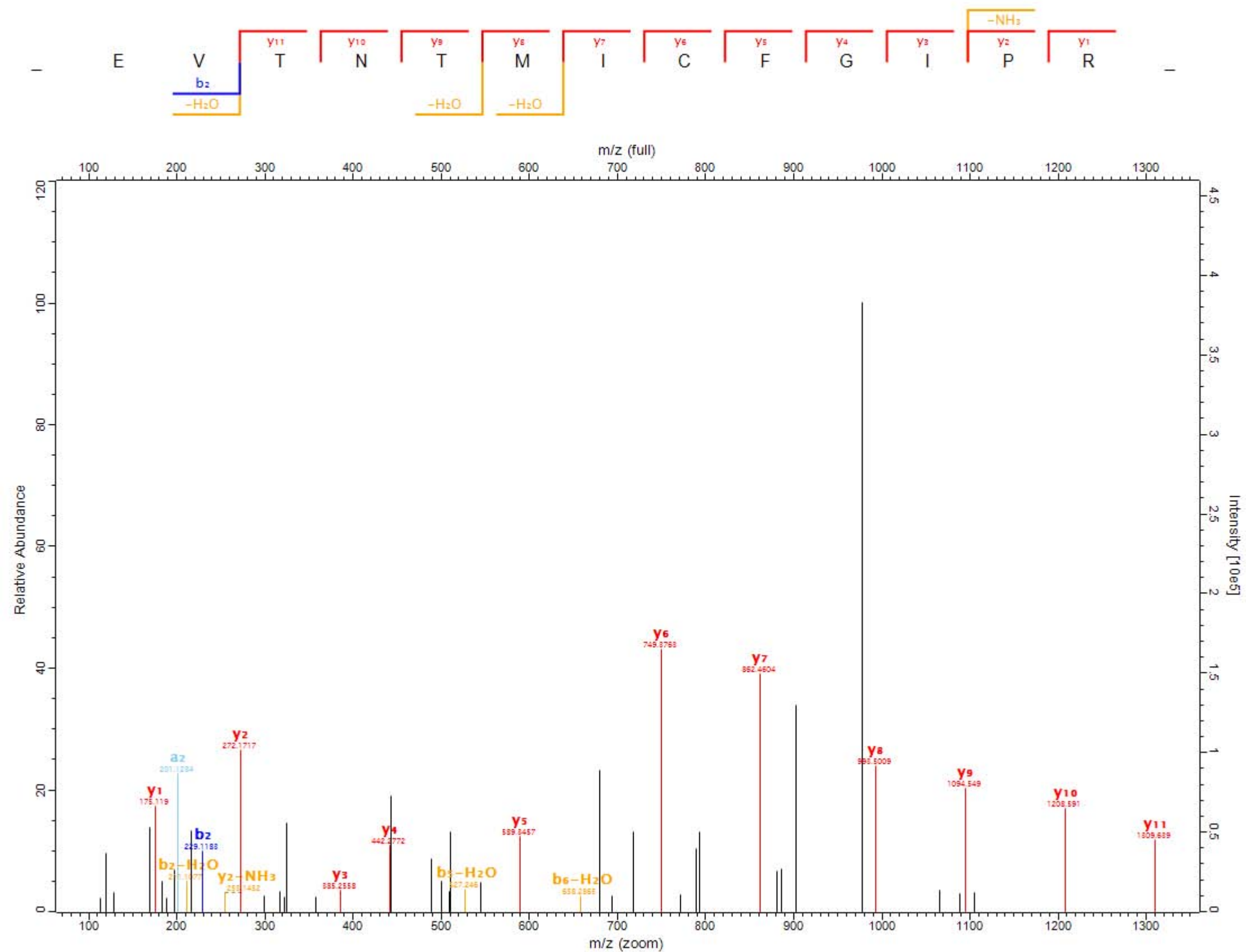

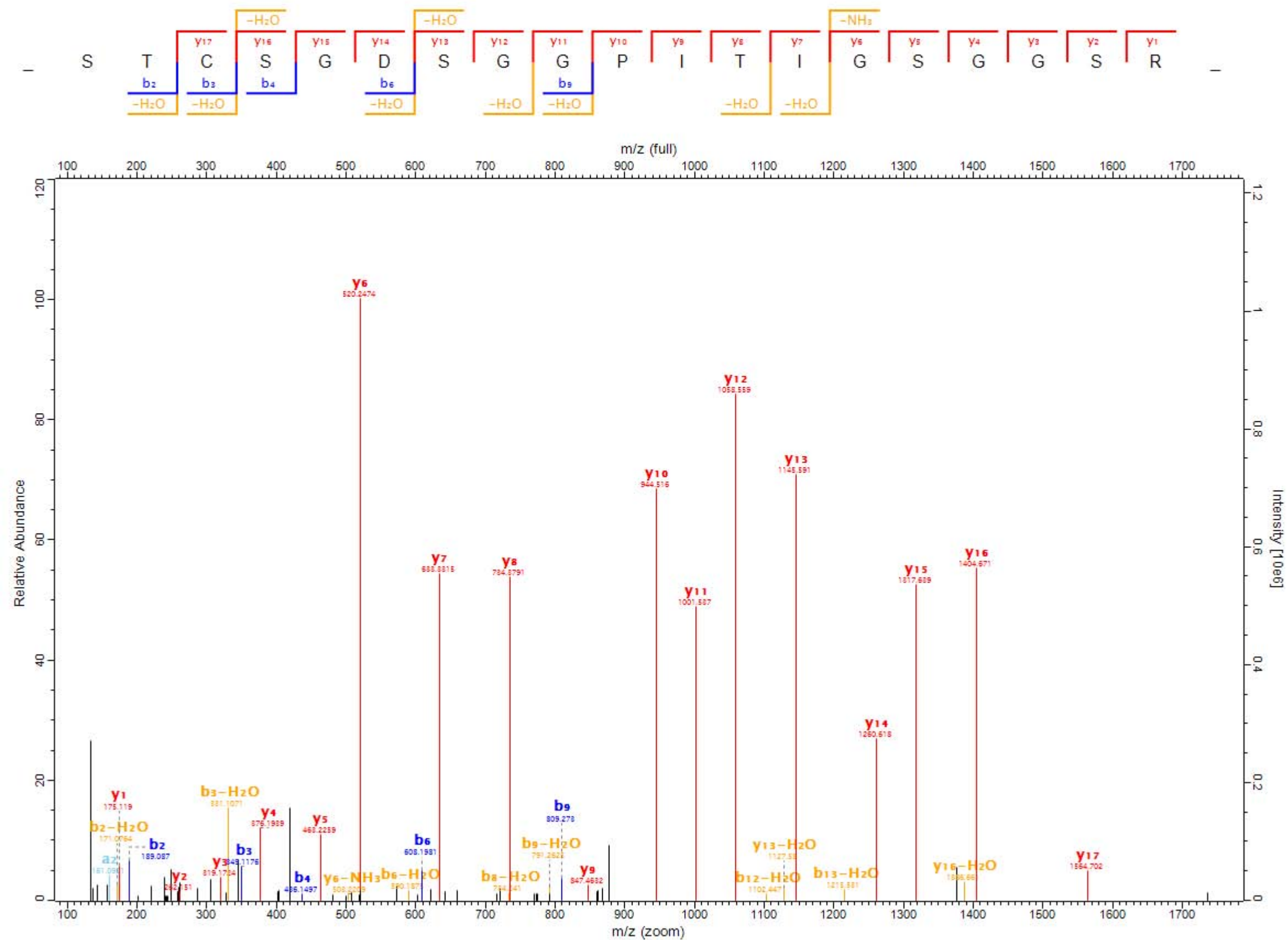

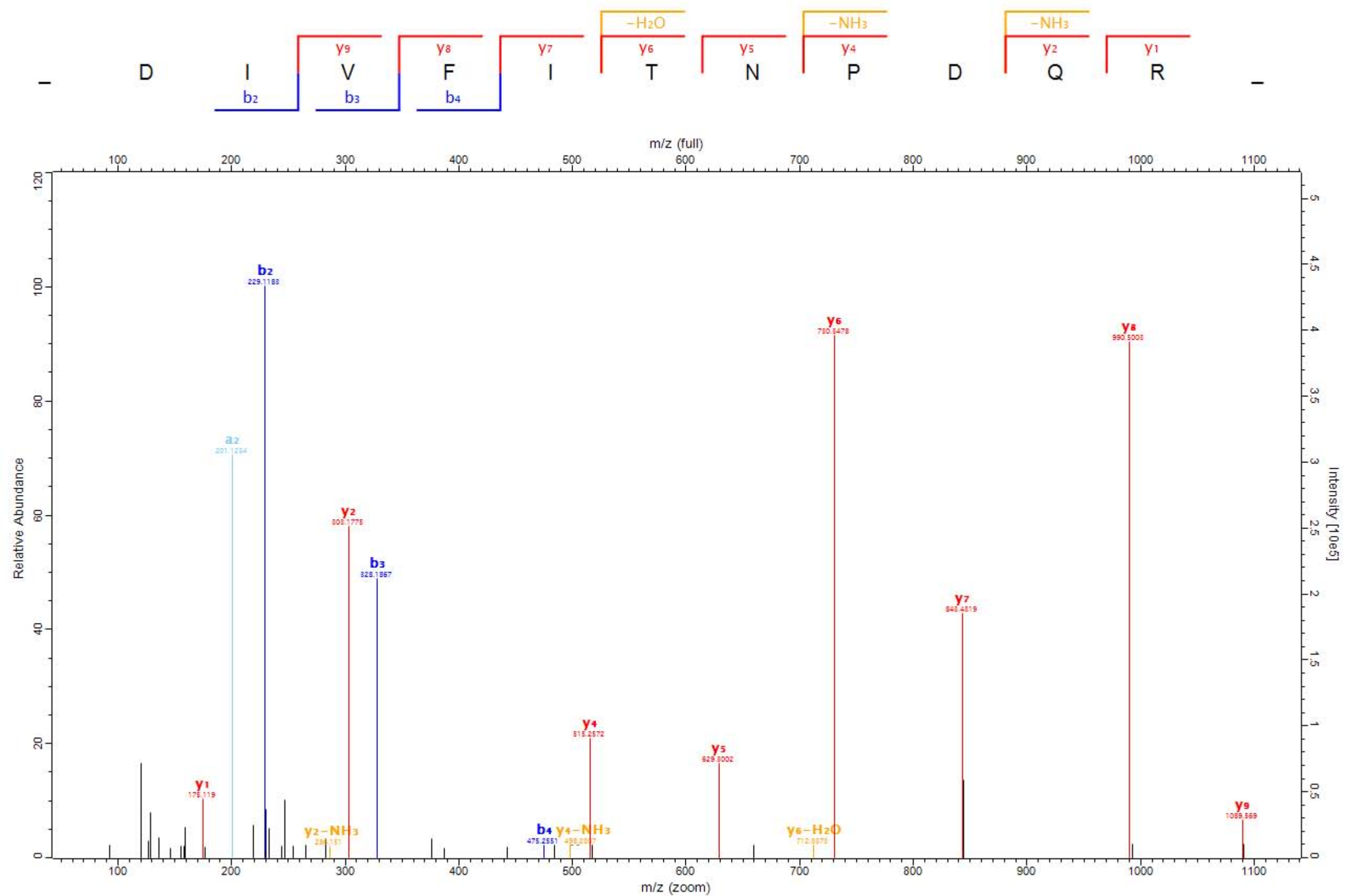

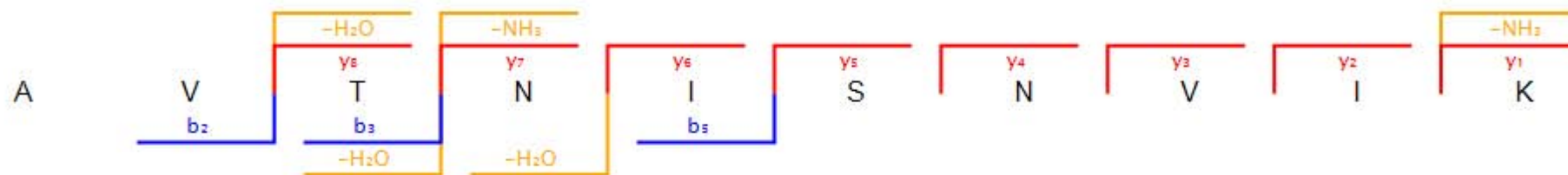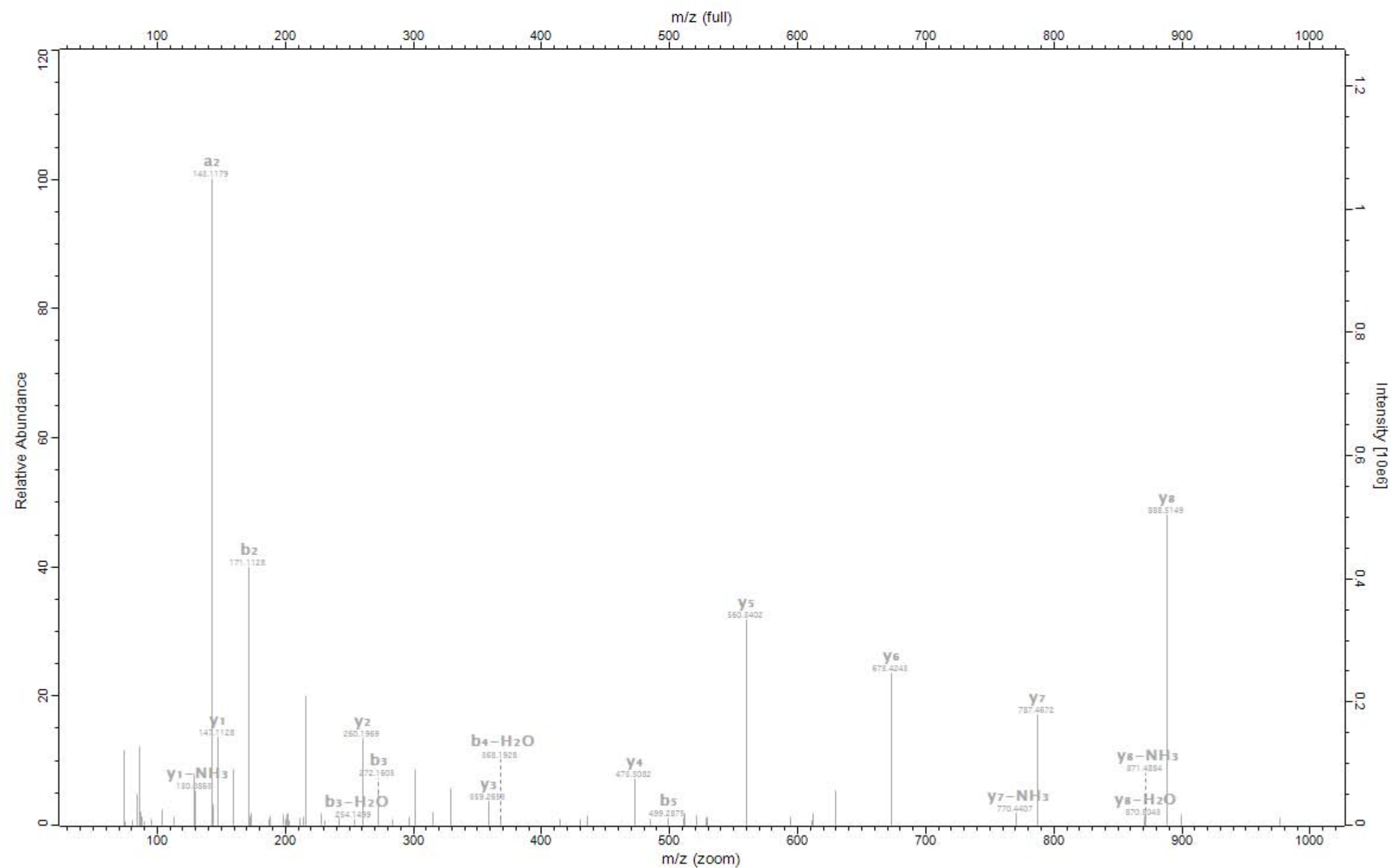

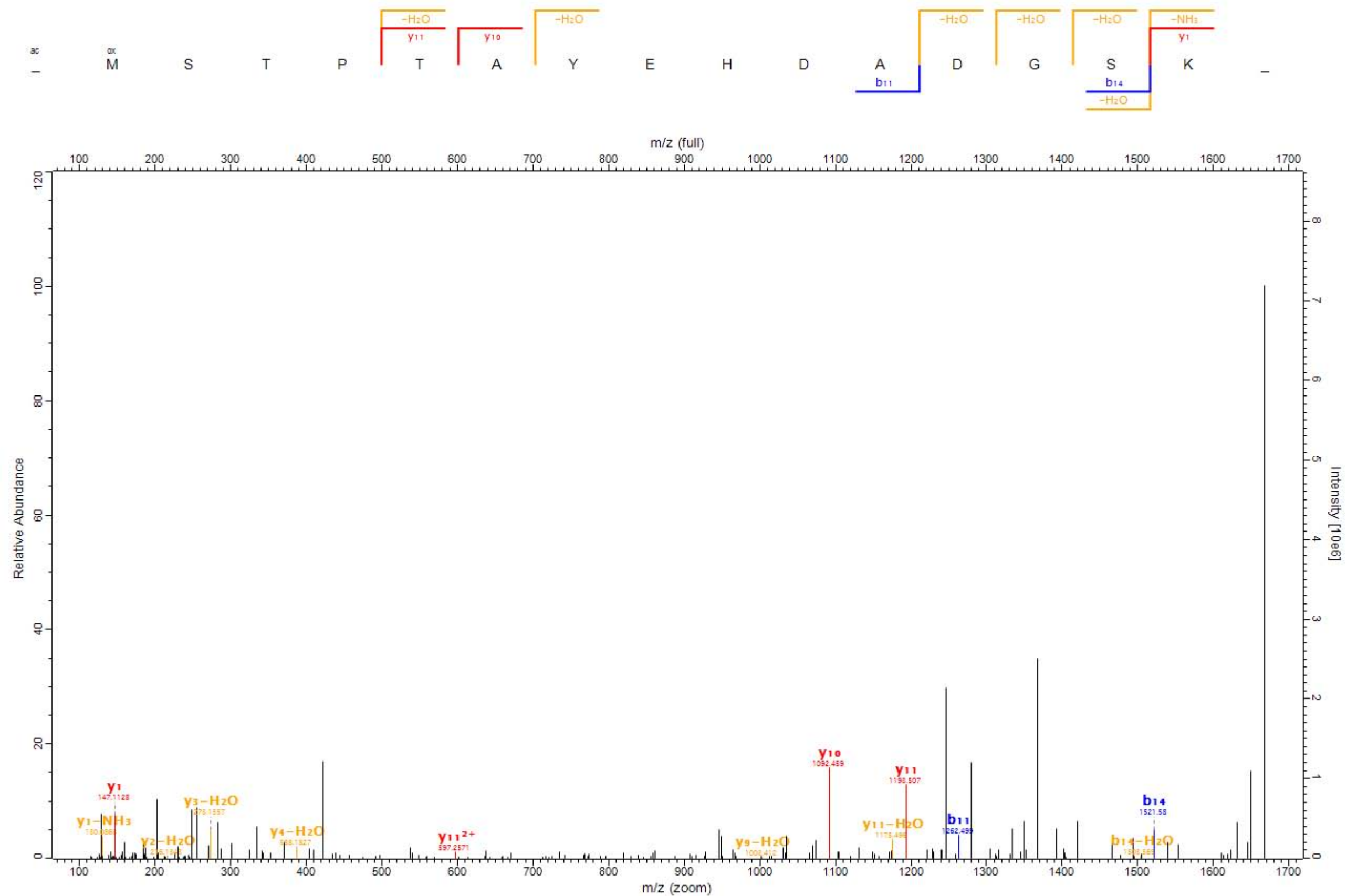

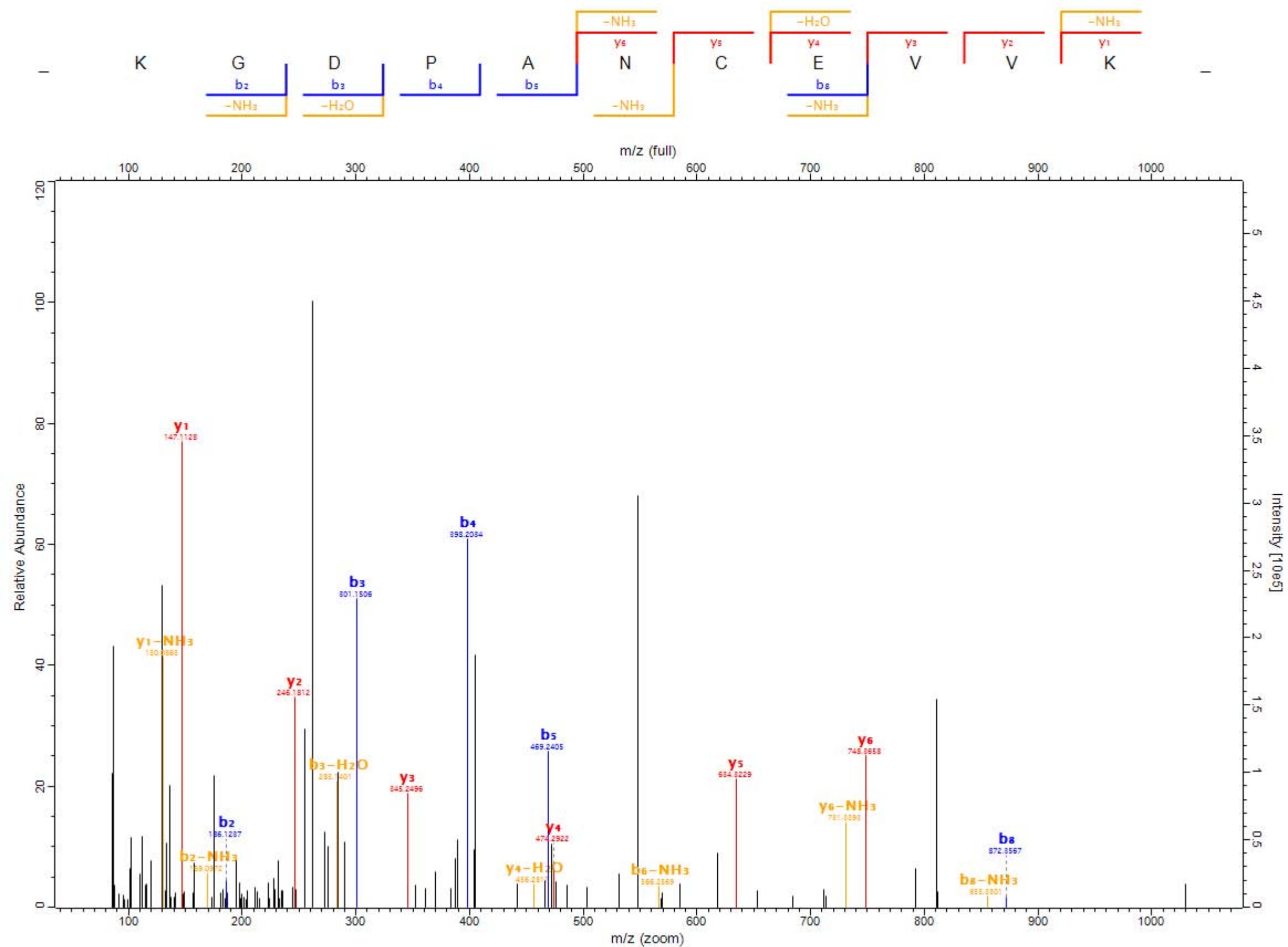

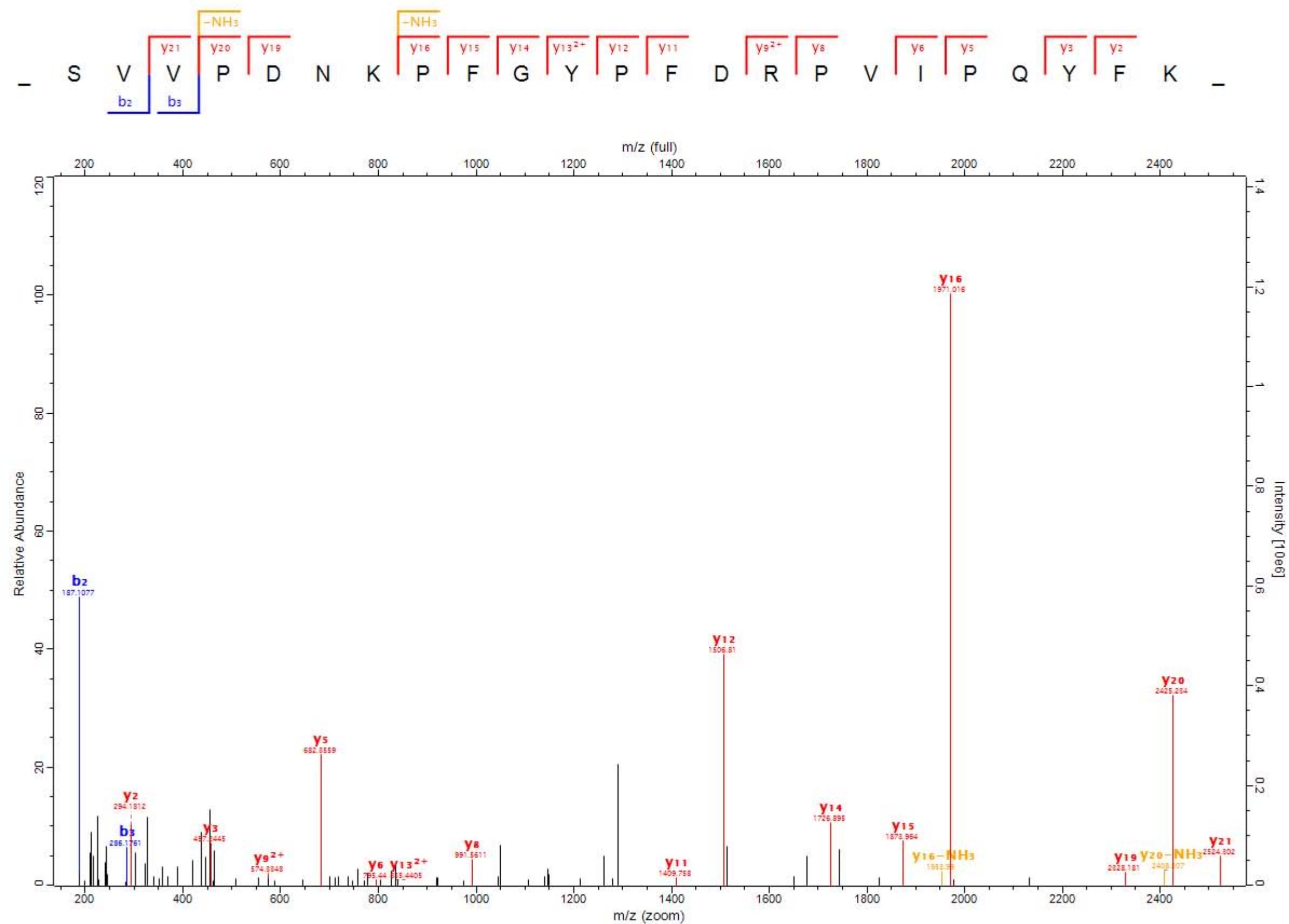

Scan number 64980 Raw file 131019jianceng1-1  
Method FTMS; HCD Peptide 98.93

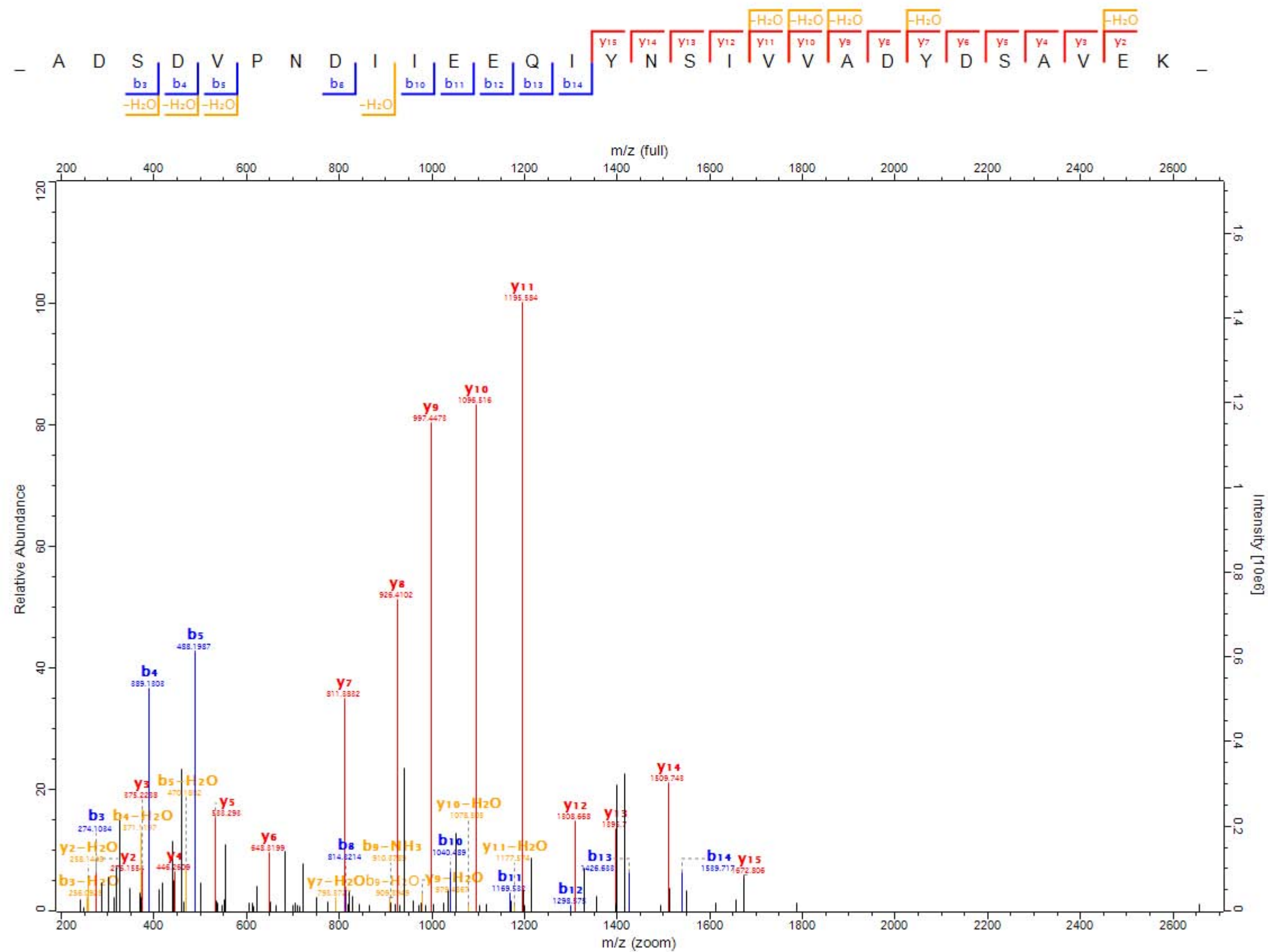

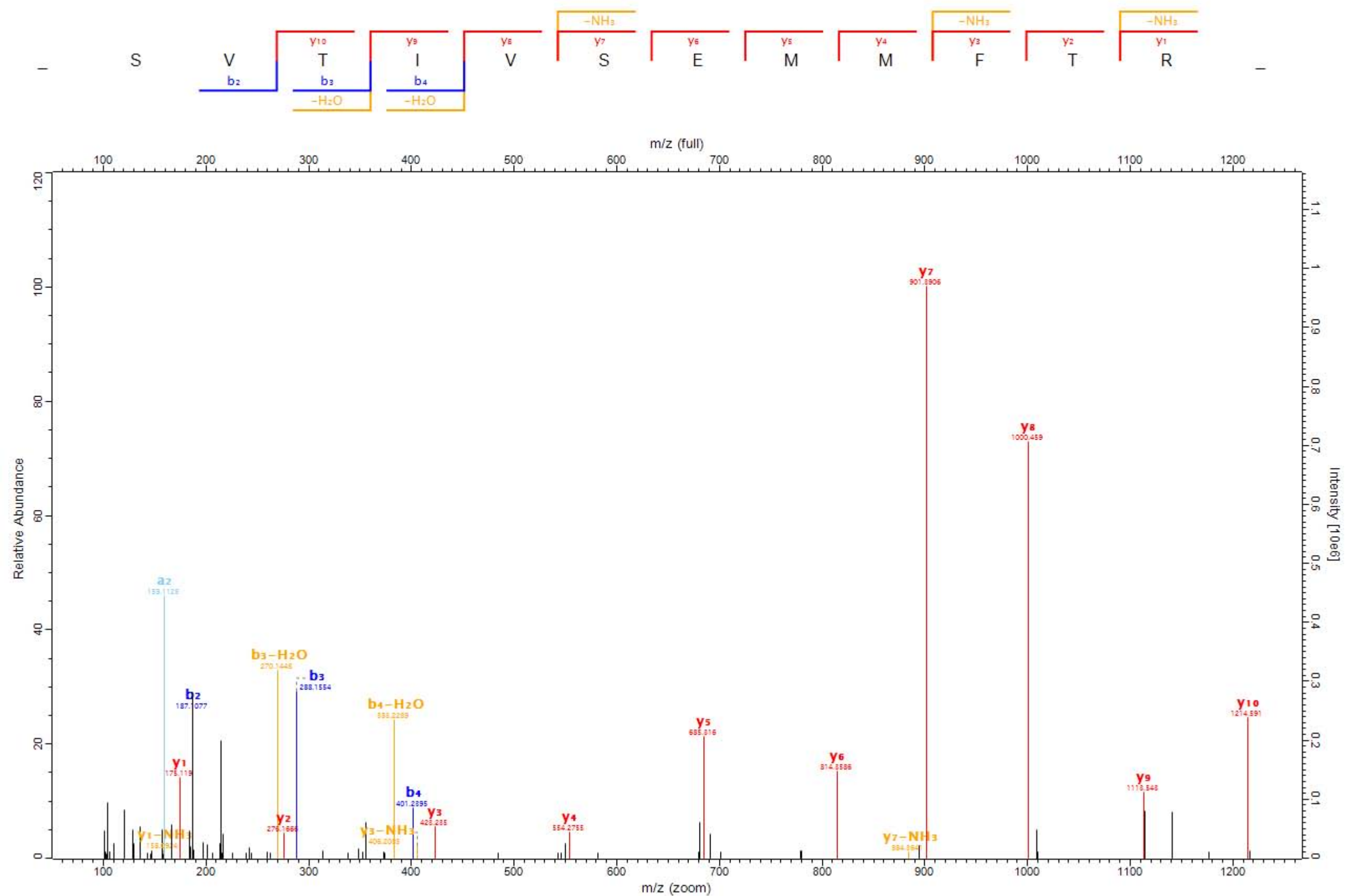

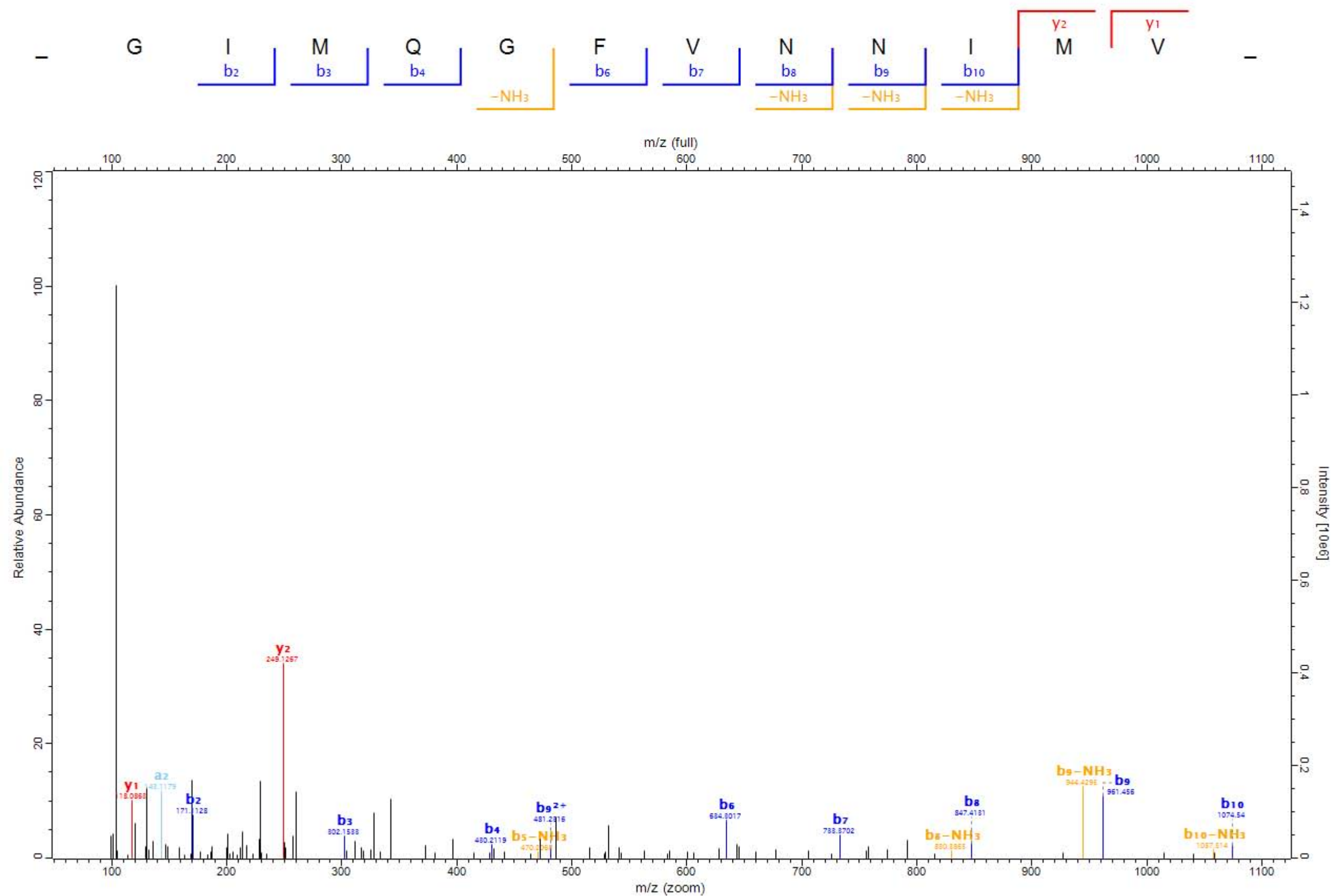

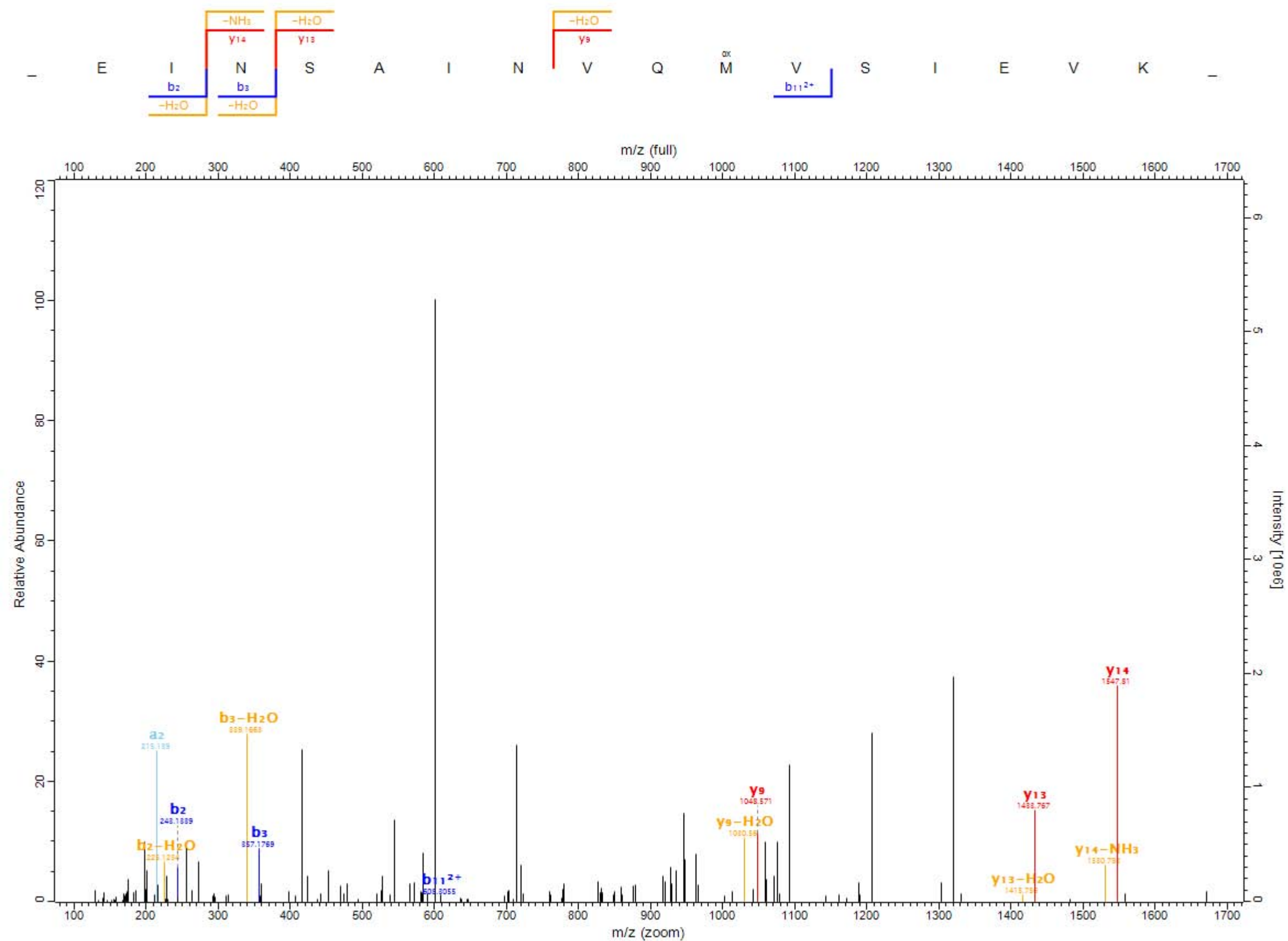

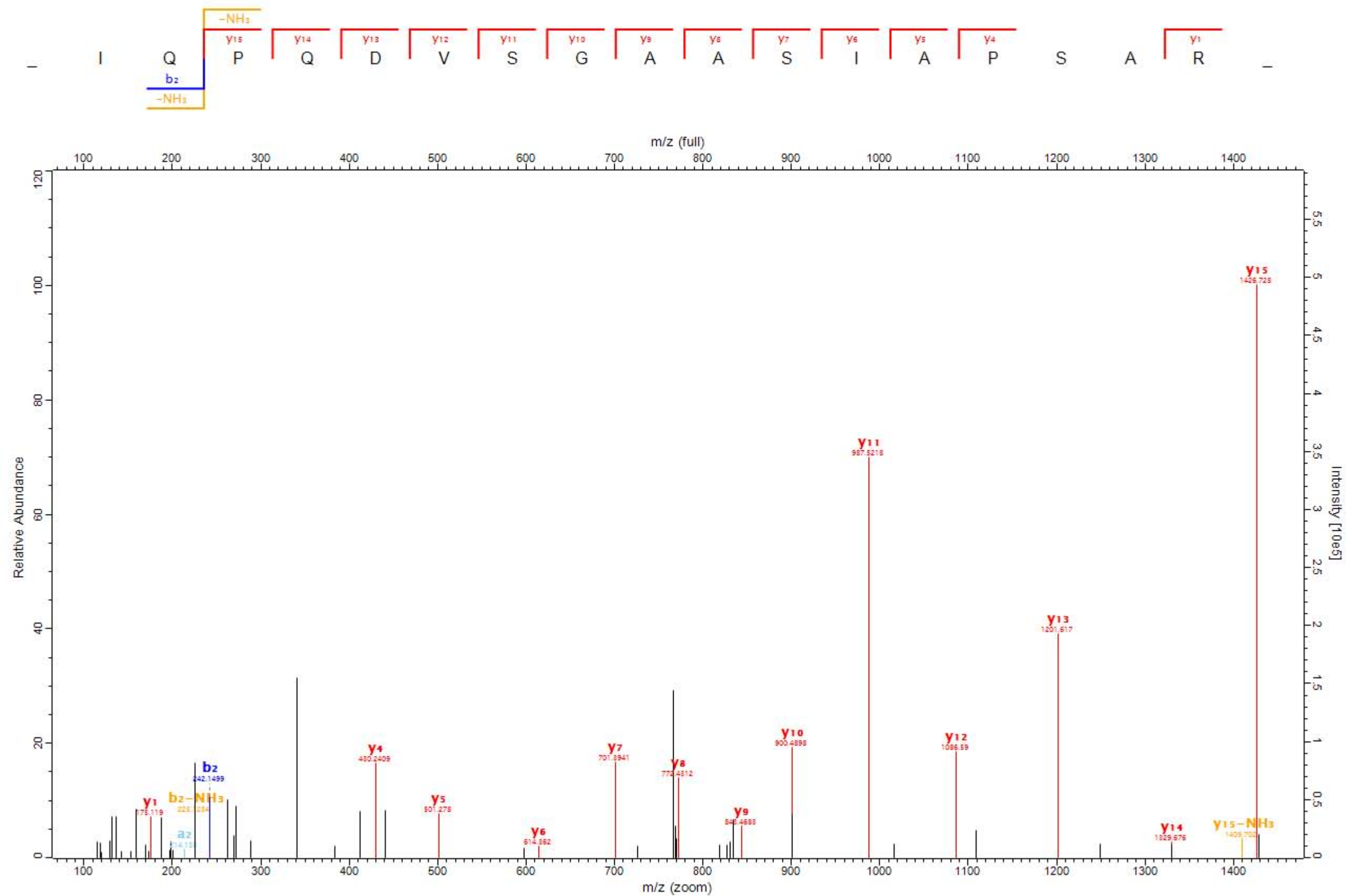

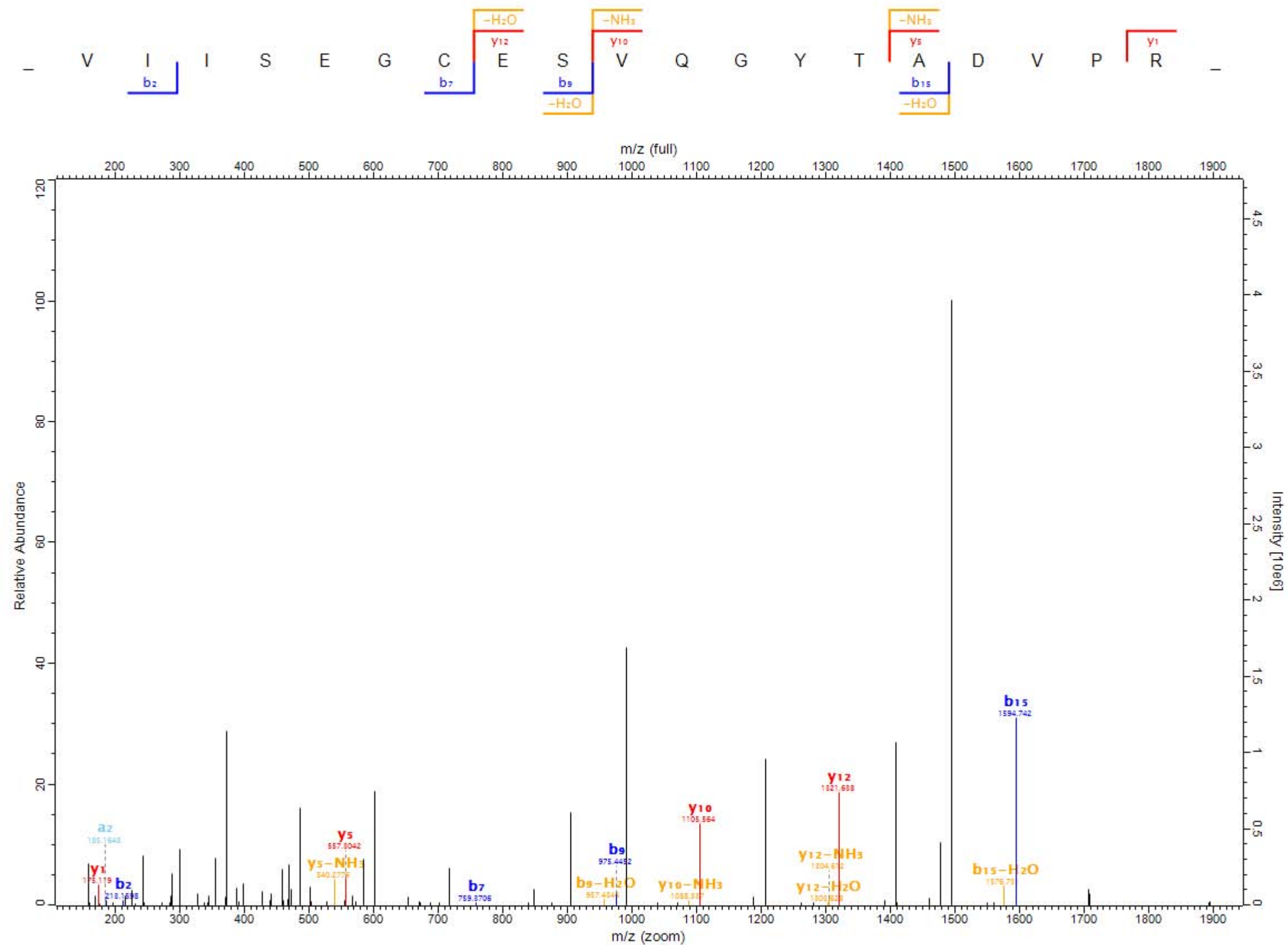

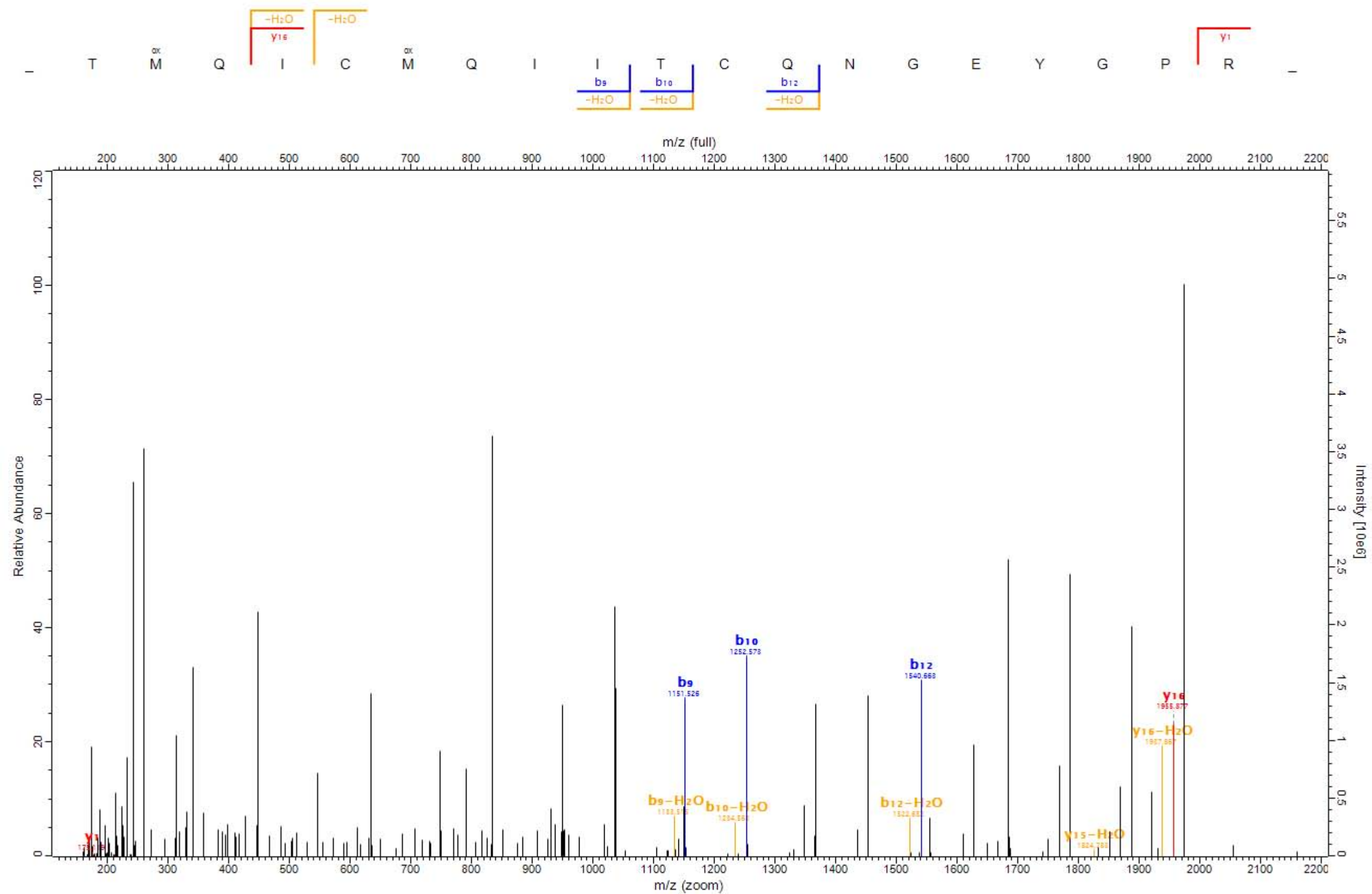

Supplement: S1 Fig — (PDF) [file pone.0123403.s001.pdf]
